# The Ampere and Electrical Standards

**DOI:** 10.6028/jres.106.005

**Published:** 2001-02-01

**Authors:** Randolph E. Elmquist, Marvin E. Cage, Yi-hua Tang, Anne-Marie Jeffery, Joseph R. Kinard, Ronald F. Dziuba, Nile M. Oldham, Edwin R. Williams

**Affiliations:** National Institute of Standards and Technology, Gaithersburg, MD 20899-0001

**Keywords:** calibration, electrical engineering, Internet, josephson arrays, measurement units, resistance measurements

## Abstract

This paper describes some of the major contributions to metrology and physics made by the NIST Electricity Division, which has existed since 1901. It was one of the six original divisions of the National Bureau of Standards. The Electricity Division provides dc and low-frequency calibrations for industrial, scientific, and research organizations, and conducts research on topics related to electrical metrology and fundamental constants. The early work of the Electricity Division staff included the development of precision standards, such as Rosa and Thomas standard resistors and the ac-dc thermal converter. Research contributions helped define the early international system of measurement units and bring about the transition to absolute units based on fundamental principles and physical and dimensional measurements. NIST research has helped to develop and refine electrical standards using the quantum Hall effect and the Josephson effect, which are both based on quantum physics. Four projects covering a number of voltage and impedance measurements are described in detail. Several other areas of current research at NIST are described, including the use of the Internet for international compatibility in metrology, determination of the fine-structure and Planck constants, and construction of the electronic kilogram.

## 1. Introduction

As a prelude to this article, we note that about 100 years before the National Bureau of Standards was created in 1901, Alessandro Volta found that an “electromotive force” (emf) was produced in an electrical circuit containing dissimilar metals and an electrolyte solution. This discovery allowed Volta to construct the world’s first electric battery. Experiments with electric current from a “Voltaic pile” led Georg Simon Ohm to discover the empirical relation known as Ohm’s law. In modern terminology [1] the equation that Ohm first published in 1826 is

$$I=\gamma \frac{A}{l}V.$$

The factor *γ* is now called the conductivity of the material, with *V* the electromotive force across a conductor of length *l* and constant cross-sectional area *A* when a current *I* flows. Ohm’s law can be written in terms of the electrical resistance *R* of a particular conductor as

V=IR.

In *Théorie mathématique des phénomènes électro-dynamiques* (1872), Andre Marie Ampère made deductions from experiments on magnetic fields and electric currents. Ampère proved that two currents produce a mutual attraction or repulsion. His formula (in present day form) for the force between two current-elements contains a constant of proportionality *μ*_0_, which is the magnetic constant (also called the permeability of vacuum). The value now assigned to this constant *μ*_0_ ≡ 4π × 10^−7^ N/A^2^ defines the ampere in such a way that electrical and mechanical measurements of quantities such as power or energy are equivalent.

### 1.1 Origins of International Electrical Units

Electrical quantities are defined by principles of electromagnetism discovered by Ampère, Gauss, Faraday, Weber, and other scientists in the first half of the 19th century. Early in the study of electrical phenomena, scientists attempted to systematize these quantities and relate them to common measurements. To compile an orderly system of such units, the British Association for the Advancement of Science established the Committee on Electrical Units and Standards in 1861. A framework for the modern electrical units was set up under the guidance of the chairman, J. Clerk Maxwell. Maxwell recognized that in both electrical and mechanical units certain quantities appear, thus these common physical units (power and energy) should represent the same unit magnitude in either system. In 1863 the committee recommended a set of “absolute practical” electrical units [2, 3] and defined their magnitudes. The set of electrical units that were selected was as follows:

ampere = unit of current,
volt = unit of emf,
joule = unit of energy,
coulomb = unit of charge,
ohm = unit of resistance,
watt = unit of power.

Metrologists then developed experimental means to standardize their electrical measurements, and an “international-reproducible” system was adopted using the best scientific measurements of the time. With respect to the ohm, the Chicago Electrical Congress of 1893 chose to recognize the resistance of a column of mercury 1.063 m long and of constant 1 mm^2^ cross section, at a temperature of 0° C as the agreed-upon 1 Ω standard. This was slightly modified for use in the United States in 1894, with the mass of the column defined as 14.4521 g, instead of defining the constant cross-sectional area [4]. The international ampere adopted at a meeting of the British Association in Edinburgh (1892) was the current that would deposit silver from a silver nitrate solution at a rate of 0.001118 g/s under specified conditions, using a silver “voltameter” [2]. These two experiments were formally recognized until the 1930s as the basis of the international ohm and ampere. Since a volt is the difference of potential produced across an ohm by a current of one ampere, only two of the three units required an experimental definition.

In 1875, the Convention of the Meter (Treaty of the Meter), a diplomatic agreement to harmonize measurements and measurement standards internationally, was signed in Paris by representatives of seventeen of the world’s most industrialized nations. The Convention established the General Conference on Weights and Measures (CGPM, a diplomatic organization), an international metrology laboratory, the International Bureau of Weights and Measures (BIPM, Bureau International des Poids et Mesures), and the International Committee for Weights and Measures (CIPM). This formal structure continues to this day as the mechanism for establishing and making changes in international measurement standards. Initially the CIPM/BIPM concentrated on mass and dimensional measurements and international electrical metrology proceeded independently, as national measurement institutes (NMIs) were created.

Following the signing of the Meter Convention, industrialized nations discovered that a need existed for national measurement laboratories, where standards could be maintained and compared, and physical constants could be determined. National standards laboratories were organized by Germany, France, and Great Britain, followed closely by the United States, in order to preserve and disseminate the units of measure. The National Bureau of Standards (renamed the National Institute of Standards and Technology, or NIST, in 1988) was created in Washington, DC, in 1901, with Division II to be devoted to electricity [4].

At the 1908 International Conference on Electrical Units and Standards in London, it was recommended that representatives of the NMIs should meet and agree on new values of international units as defined by the mercury ohm and silver voltameter. In April and May 1910, the International Technical Committee met in Washington, DC at NBS. Scientists from Germany, France, and Great Britain brought standard resistors and standard cells that had been carefully evaluated in terms of their national units. Dr. E. B. Rosa was the first Chief of the Electricity Division and headed this committee. Comparisons made at the meeting showed that the resistance unit represented by the German standards was only 1 ×10^−5^ larger than that of the British. Results of work then in progress under Dr. Rosa were in reasonable agreement, and the committee recommended that all countries use, as the international ohm, the mean of the values found by Germany and Great Britain [2, 5]. Using the average emf obtained with silver voltameter experiments from the four nations, the Committee also recommended that the voltage of the Weston normal cell (described in Sec. 1.3.2) at 20° C be assigned a value of 1.0183 international volts. The precision of the measurements was somewhat higher, however, with the average international value being 1.018312 (8) V [5]. On January 1, 1911, the system of “international units” was inaugurated, based on the Committee results. As a byproduct of the work of the International Technical Committee of 1910, NBS came into possession of a group of 36 standard cells, part of the large group of Weston cells that had been used to assign this value to the Weston Normal Cell [2].

### 1.2 Movement Towards Absolute Electrical Units

In 1921, the Sixth CGPM modified the Meter Convention to extend the scope of the BIPM and CIPM to cover electrical measurements. This decision was implemented in 1927 with the formation of the Consultative Committee on Electricity (CCE), an advisory body of international experts, which formulated the responsibilities of the BIPM in the area of electricity. The program went into effect in 1929.

The CIPM decided in 1929 that it was no longer possible to maintain the international units with the degree of accuracy demanded by science using the existing “international” definitions. The national laboratories were finding it more and more difficult to maintain the international units based on independent measurements of the mercury ohm and silver ampere. Problems as fundamental as the existence of silver isotopes were known to cause errors. In addition, the corrections that had to be applied for the difference between international and absolute units were becoming appreciable in calorimetry, thermometry, and measurements of fundamental constants [2]. At the Eighth CGPM in 1933, the consensus among representatives was that new types of “absolute ohm” and “absolute ampere” experiments should be used to define the three main electrical units. These experiments would be based on the interactions between mechanical and electrical phenomena. As Maxwell had pointed out, this would make electromagnetic quantities—such as the ampere, volt, and ohm—fully consistent with other units in science and engineering. There were already in existence several promising methods for relating these quantities to the mechanical units of length, time, and mass. Since the experiments required careful electrical and dimensional measurements as well as complex calculations, the new “absolute determinations” would be pursued primarily at the large NMIs.

#### 1.2.1 Development of Absolute Determinations

H. L. Curtis and R. W. Curtis published “An Absolute Determination of the Ampere” in the Bureau of Standards Journal of Research in 1934 [6]. This measurement used a Rayleigh current balance, in which the electromagnetic force between concentric coils is balanced by the gravitational force on a mass. Within 4 years, two absolute-ohm determinations were completed by H. L. Curtis, C. Moon, and C. M. Sparks [7, 8]. They computed the value of inductors in absolute units from dimensional measurements and from the permeability of the surrounding medium, and measured those values in international units, based on fixed standards of resistance.

**Fig. 1:**
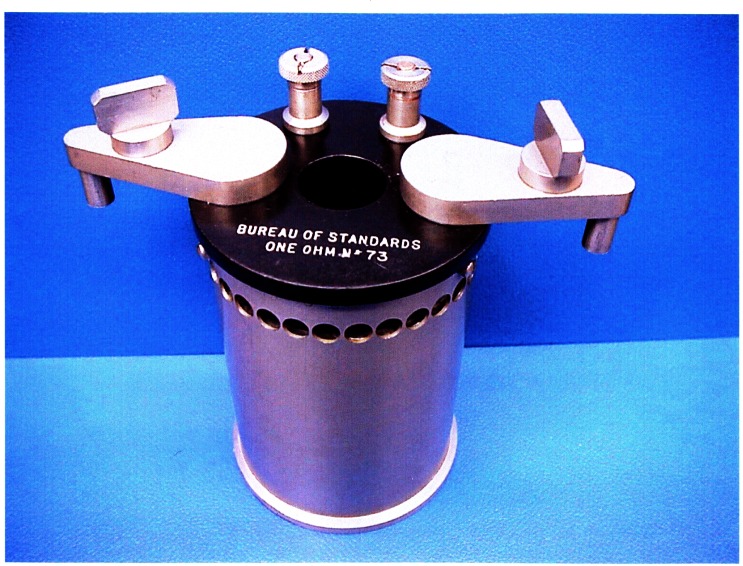
A double-walled 1 Ω standard resistor of the Thomas type.

In the 1920s, Dr. James L. Thomas had taken up the task of improving the long-term stability of wire-wound resistors, which were used to measure the current in absolute determinations. When a resistor is made by winding wire on a spool, parts of the crystalline structure of the wire are stressed past their elastic limit. Thomas developed wire-wound standard resistors that were annealed at high temperature, which released some of the internal strains and reduced the rate of change of resistance with time [1]. Heat-treated manganin wire resistors developed by Thomas incorporated hermetically-sealed, double-walled enclosures, with the resistance element in thermal contact with the inner wall of the container to improve heat dissipation. These 1 Ω Thomas-type standards [9, 10] proved to be quite stable with time, and quickly came into favor as the primary reference for maintaining the resistance unit at NBS and at many other NMIs (see Fig. 1).

**Fig. 2:**
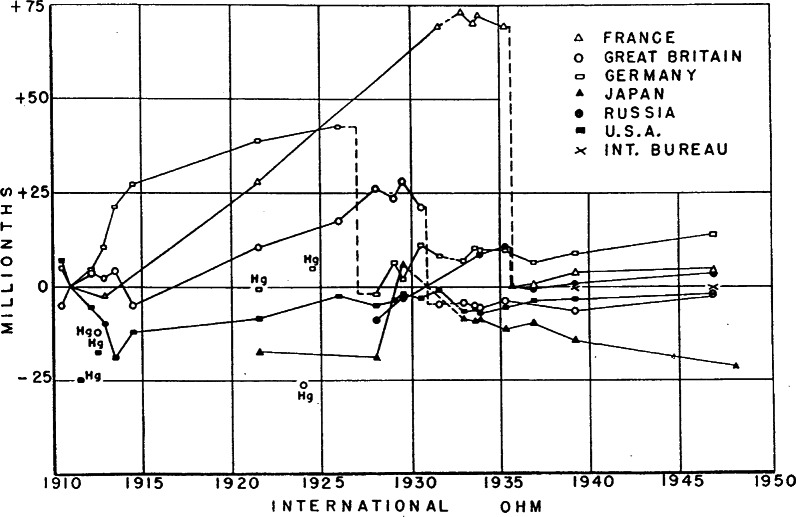
Differences from the adjusted mean values of the International Ohm, as maintained by various national laboratories, 1911 to 1948. Points marked “Hg” indicate the results of measurements on mercury columns. Reprinted from Ref. [2].

By 1944, three absolute-ampere and three absolute-ohm experiments were completed at the Bureau [11], and similarly accurate absolute determinations of the ampere and ohm were available from Britain. At that point (see Fig. 2), according to H. L. Curtis [11], “during the last two decades not one of the national laboratories has reestablished its unit for the international ohm by making measurements upon the mercury ohm. Instead each laboratory maintains its international ohm by means of wire resistance standards.” Thus, after World War II, the CIPM approved long-awaited new recommendations for the electrical units. These values were based on the absolute determinations, and superceded the mercury ohm and silver ampere on January 1, 1948 [2]:

1 mean international ohm = 1.000 49 (pre-1948) absolute ohm;
1 mean international volt = 1.000 34 (pre-1948) absolute volt.

#### 1.2.2 The Absolute Ohm

**Fig. 3:**
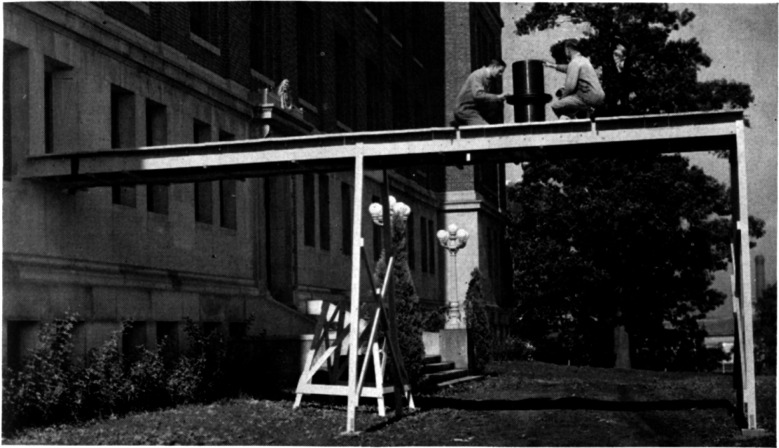
A photograph of the outdoor pier with a wooden model showing the location of the mutual inductor, from the 1949 report “An absolute measurement of resistance by the Wenner method” [16].

Work continued on improving the absolute measurements of electrical units and, in 1949, J. L. Thomas, C. L. Peterson, I. L. Cooter, and F. R. Kotter published a new measurement of the absolute ohm [12] using an inductor housed in a non-magnetic environment (see Fig. 3). Using the Wenner method of measuring a resistance in terms of a mutual inductance and a rate of rotation, their work gave a value of 0.999 994 absolute ohm for the new as-maintained unit of resistance at NBS. The mean value assigned to ten Thomas-type standard resistors from this experiment was found to have been the same between 1938 and 1948 to within 1 μΩ/Ω. Thomas et al. wrote in a 1949 paper, this was “the first satisfactory method that has been devised for checking the stability of the unit as maintained by a group of wire-wound resistors.”

In 1956, Thompson and Lampard at the Australian national metrology laboratory determined that the value of a special type of cross-capacitor was dependent only on the length of the capacitor, the speed of light, and the permeability of free space. Based on this principle, the calculable cross-capacitor could provide an alternative method of evaluating the unit of resistance based on straightforward measurements of length and time. R. D. Cutkosky developed this absolute capacitance standard and the complex impedance-bridge techniques needed to transfer the results to the ohm unit maintained at NBS [13]. Cutkosky obtained the value of the U.S. Legal Ohm in absolute units with a relative standard uncertainty of 2.1 μΩ/Ω in 1961, using gauge bars as electrodes for the capacitor. This measurement [13, 14] indicated that the value of the NBS unit of resistance was 1.000 000 6 Ω on that date. A more precise cross-capacitor constructed in the late 1960s has yielded a number of improved values of the farad, the ohm, and of related fundamental constants. In 1974, Cutkosky’s calculable capacitor chain (see Sec. 2.3.3) yielded a value of the ohm with a relative standard uncertainty of 0.03 μΩ/Ω.

#### 1.2.3 The Absolute Ampere

Improved absolute measurements of current were in some ways more difficult than those of the ohm, and proceeded by smaller steps. Before WW II, at about the same time that the moving-coil current balance was being used to determine the ohm, H. L. Curtis and R. W. Curtis had started to prepare a balance of a special design for the absolute ampere determination. In 1958 R. L. Driscoll reported results from this Pellat balance [15]. The mechanical measurement was of the torque on a small coil, with axis at right angle to the magnetic field of a large horizontal solenoid. When the current passing through the small coil was reversed, it produced a force that could be balanced by a mass of about 1.48 g placed on the balance arm. The large stationary coil was wound on a fused-silica former and the balance beam was equipped with knife-edges and supports machined from natural agate. The effect of the measured dimensions of the small coil on the computed mutual inductance was the largest contribution to the uncertainty, which totaled about 8 μA/A. Also contributing to the uncertainty were the determination of the balancing mass and of the acceleration due to gravity.

As soon as possible after completing the Pellat balance measurement, Driscoll and Cutkosky [16] repeated the 1934 Rayleigh current-balance determination of the ampere using the original apparatus. The results of these two experiments, 1 NBS ampere = 1.000 013 ± 0.000 008 absolute amperes by the Pellat method, and, 1 NBS ampere = 1.000 008 ± 0.000 006 absolute amperes by the current balance, were in good agreement. This gave an overall relative uncertainty in the ampere at NBS of about 5 μA/A at that time (1958), and verified that the ratio of emf over resistance of the maintained standards had been constant to within about one part in 10^5^ since 1942.

### 1.3 Modern Electrical Standards at NIST

#### 1.3.1 The Representation and Maintenance of the Ohm

The value of the U.S. representation of the ohm or “legal” ohm maintained at NIST has been adjusted only twice. This occurred once in 1948 when the ohm was reassigned using a conversion factor relating the international reproducible system of units [3] to the precursor of the International System of Units (SI) derived from the fundamental units of length, mass, and time. The second reassignment was in 1990 when the ohm became based on the quantum Hall effect (QHE) described in Sec. 1.4.2. After 1960, ohm determinations were made using calculable capacitors based on the Thompson-Lampard theorem and a sequence of ac and dc bridges (see Sec. 2.3). Then came the discovery of the QHE in 1980, which has provided an invariable standard of resistance based on fundamental constants. Consequently, on January 1, 1990 the U.S. Legal Ohm was re-defined in terms of the QHE, with the internationally-accepted value of the quantum Hall resistance (or von Klitzing constant, after the effect’s discoverer) based on calculable capacitor experiments and other fundamental constant determinations. At that time, the value of the U.S. Legal Ohm was increased by the fractional amount 1.69 × 10^−6^ to be consistent with the conventional value of the von Klitzing constant [17].

From 1901 to 1990, the U.S. Legal Ohm was maintained at 1 Ω by selected groups of manganin resistance standards [18]. Four different types of resistance standards have been represented in these groups, whose numbers have varied from 5 to 17 resistors. From 1901 to 1909, the group comprised Reichsanstalt-type [19] resistance standards made by the Otto Wolff firm in Berlin. These standards were not hermetically sealed and consequently underwent changes in resistance as a function of atmospheric humidity. In 1907 Rosa cured the problem by developing a standard whose resistance element is sealed in a can filled with mineral oil [20]. The U.S. representation of the ohm was maintained by ten Rosa-type 1 Ω resistance standards from 1909 to 1930. Over the years, measurements of differences between the individual Rosa-type resistors indicated that the group mean was probably not constant. Thomas in 1930 reported on the development of his new design for a resistance standard having improved stability [9]. The Thomas resistance standards were more stable immediately following construction than the Rosa-type resistors and two were added to the primary group in 1930. Eventually, in 1932, the Rosa-type resistors in the primary group were replaced by the Thomas resistors. To reduce loading errors, Thomas in 1933 improved the design of his resistor by using manganin wire of larger diameter mounted on a larger diameter cylinder to increase the dissipation surface area [10]. A select group of the new-design Thomas resistors was used to maintain the U.S. Legal Ohm from 1939 until its re-definition in 1990 based on the QHE.

#### 1.3.2 The Representation and Maintenance of the Volt

For almost 80 years starting in 1901, the U.S. Legal Volt was maintained by several groups of standard cells. There was a large effort in the late nineteenth century and the early twentieth century to establish a standard for electromotive force (emf) based on electrochemical reactions within chemical cells. The first legal unit of voltage for the United States was based on the Clark cell, developed by Latimer Clark in 1872 [21], with its output assigned a value of 1.434 international volts by the 1893 International Electric Congress. Public Law 105, passed by the U.S. Congress in 1894, made this the legal standard of voltage in the U.S. During the years between 1893 and 1905, the standard cell devised by Edward Weston was found to have many advantages over the Clark cell [2]. The Weston cell consists of a cadmium amalgam anode and a mercury-mercurous sulfate cathode with a saturated cadmium sulfate solution as the electrolyte. In 1908 at the London International Conference on Electrical Units and Standards, the Weston cell was officially adopted for maintaining the volt. After 1908 only Weston cells were used for maintaining the national standard in the United States.

The Weston standard cell can be disturbed by transport or if subjected to a change in temperature or a small electrical current. When at times it was necessary to eliminate cells—due to changes in emf of a cell relative to the mean of the group—new cells could be added. In 1965 the National Reference Group of standard cells [22] included eleven cells made in 1906, seven cells made in 1932, and 26 cells made in 1948. Long-term stability of the volt reference was also maintained by comparisons of neutral and acid cells, preparing and characterizing new cells, and through international comparisons and absolute ampere and ohm experiments. According to Driscoll and Olsen [23], the results of the absolute current balance measurements could be regarded “as assigning a value to the emf of the standard cell used to control the strength of the current” and as a check on the emf of the NIST standard cell bank. The use of the Weston cell as the national standard of voltage was supported by a considerable amount of research in electrochemistry and related fields at NBS as seen by the staffing below.

**Table:** 

Performance of standard cells	R. J. Brodd, W. G. Eicke. H. E. Ellis, C. E. Waters, F. A. Wolfe
Chemistry and thermodynamics	L. H. Brickwedde, D. N. Craig, W. J. Hamer, P. E. Robb, G. W. Vinal, F. E. Vinal
Experimental cells	V. E. Bower, W. J. Hamer, C. A. Law, G. Roberts, A. S. Skapars, G. W. Vinal
Instrumentation	H. B. Brooks, W. G. Eicke, B. F. Field, F. K. Harris, P. A. Lowrie, B. A. Wyckoff

Work in this area through 1964 is summarized in NBS Monograph 84 [22].

Before the Josephson effect was discovered (see Sec. 1.4.1), it was difficult to provide incontrovertible evidence regarding the long-term stability of the U.S. Legal Volt. However, considerable evidence indicated that the unit of emf preserved with standard cells was unlikely to have changed by any significant amount, relative to the best measurements of the time, from the early 1900s to the 1960s.

In the late 1950s, research in solid-state physics stimulated the growth of the semiconductor industry. A new type of voltage standard based on a solid-state device, the Zener diode, appeared in the early 1960s. W. G. Eicke at NBS first reported the possibility of using Zener diodes as transport standards [24]. In the following years, after several manufacturers started making commercial Zener voltage standards, these references began to replace standard cells in commercial use. Although Zener voltage standards exhibit higher noise characteristics than standard cells, and are affected by environmental conditions of temperature, atmospheric pressure, and relative humidity, they are now widely used in many metrology labs because of their robust transportability.

#### 1.3.3 Physical Constants

In the early 1950s, director E. U. Condon supported a greater emphasis on physics and basic standards at NBS. The Electricity and Optics Division began preparations for measuring the fundamental constant *γ*_p_, the gyromagnetic ratio of the proton, which would relate the measurement of magnetic field strength to the nuclear magnetic resonance (NMR) frequency of the proton. This measurement grew out of the work on the measurement of relative positions of currents in precision solenoids used in the ampere determinations. Driscoll and Olsen noted [23], “it was kept in mind during the construction that (the Pellat) solenoid might be used to provide a magnetic field for a later measurement of *γ*_p_.” The single-layer solenoid together with a set of Helmholtz coils did provide a uniform field region suitable for the NMR measurements used to measure *γ*_p_ (denoted by *γ′*_p_ in water samples). Early measurements were made by the method of free precession, and later ones were done by the nuclear induction method. These measurements yielded a final result in 1979 [25], with a relative standard uncertainty of about 2.1 × 10^−7^.

An entirely new apparatus was begun thereafter to measure *γ′*_p_ in low magnetic field, and to help test the theory of the Josephson effect, quantum electrodynamics, and the newly discovered quantum Hall effect. The improvements in this apparatus included a method of injecting compensation currents into selected solenoid windings, which reduced the need for measurements of the mean solenoid diameter [26]. The dimensional measurements, the NMR measurements, and the various calibrations contributed about 0.5 × 10^−7^ each in relative uncertainty. The result, for protons in a spherical sample of water at 25 °C, was

$$\gamma_{p}^{\prime }\left(\text{low}\right)=2.67\;513\;376\times 10^{8}s^{-1}{T^{-1}}_{\text{NIST}},$$

with a total relative uncertainty of 1.1 × 10^−7^. The flux density was measured in terms of the NIST laboratory units of resistance and voltage at the time.

#### 1.3.4 1990 Practical or Laboratory System of Units

NIST determinations of physical constants, namely *γ′*_p_ [26], the Josephson frequency-to-voltage ratio 2*e*/*h* [27], the quantized Hall resistance (QHR) in terms of a resistance derived from the calculable capacitor [28, 29], and the moving-coil force-balance determination of the watt [30], were analytically combined [31] in 1989 to obtain values of certain fundamental constants. These results made a significant contribution to the 1990 recommended values of the Josephson and von Klitzing constants that were used to establish laboratory representations of the volt and ohm (based on the Josephson and quantum Hall effects) that were consistent with the SI. By international agreement, Josephson and QHE devices are now the preferred reference standards for measurements of voltage and resistance. A third quantum physics effect, based on “single electron tunneling” (SET), could lead to an absolute electron-counting device based on quantum physics and further improvements for the units farad and ampere. Contributions to the physical and metrological understanding of these quantum-effect devices are discussed in the following section.

### 1.4 Quantum Physics and Nobel Prizes

Two Nobel Prize-winning discoveries in condensed matter physics, the Josephson effect [32] by Brian D. Josephson in 1962 and the QHE [33] by Klaus von Klitzing in 1980, have provided significant improvements in how the SI volt and ohm are maintained and disseminated. This section describes the early development of these two effects into intrinsic voltage and resistance standards whose values, under proper operating conditions, depend only on fundamental constants of nature.

#### 1.4.1 The Josephson Effect

**Fig. 4:**
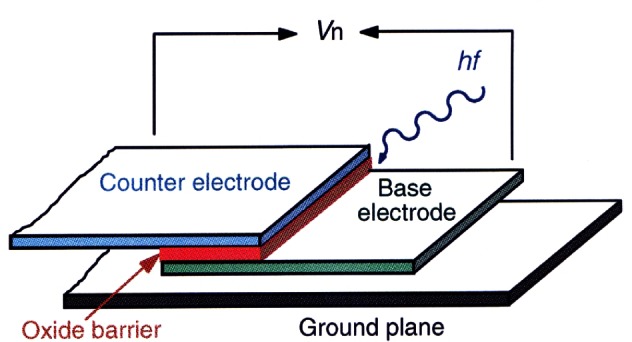
Josephson junction structure irradiated by microwaves of energy *hf*, producing a dc voltage *V*_n_ across the junction.

In 1962, Brian Josephson, a graduate student at Trinity College, Cambridge, England, predicted [32] that electrons can tunnel in pairs (Cooper pairs) between two superconductors separated by a thin insulating barrier (a weak link or Josephson junction). An applied dc voltage *V* across the barrier would generate an ac current at the frequency *f* = 2*eV*/*h*, where *e* is the elementary charge and *h* is the Planck constant. Conversely, an applied ac current of frequency *f* would generate a dc voltage *V_n_* (see Fig. 4) at the quantized values

$$V_{n}=nh\;f/2e,$$ (1)

where *n* is an integer and the value of 2*e*/*h* is approximately 483.6 MHz/μV. Josephson wrote his Ph.D. thesis on this theory [34], which won a share of the 1973 Nobel Prize in physics.

**Fig. 5:**
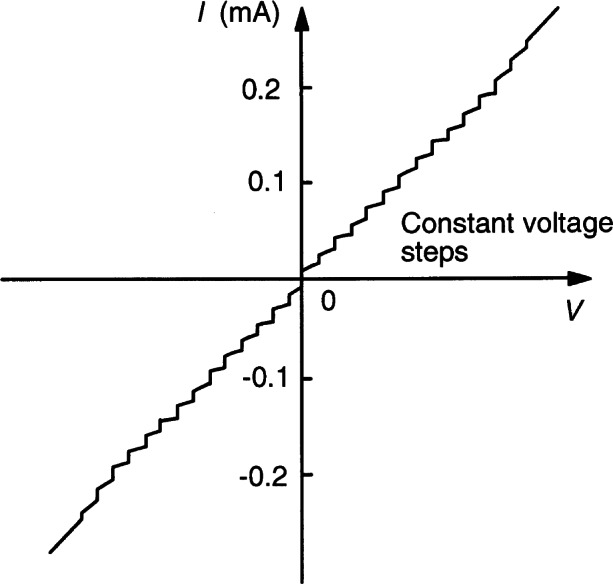
A series of steps of constant voltage generated by a Josephson junction array.

Anderson and Rowell [35] provided the first experimental verification of the theory by observing a dc current (a supercurrent) across a tunnel barrier with no applied dc voltage. Shapiro [36] then obtained the constant voltage steps *V_n_* of Eq. (1) by using rf microwaves to generate an ac current of frequency *f* across the superconductors (see Fig. 5 for an example). Clark [37] showed in 1968 that the value of 2*e*/*h* obtained from Josephson effect measurements was material-independent to within 1 part in 10^8^ by applying the same microwave radiation to pairs of dissimilar Josephson junctions and comparing the junction voltages. In 1968, Parker, Langenberg, Denenstein, and Taylor [38] compared, via a potentiometer, the Josephson voltages of junctions consisting of five different superconducting materials and various combinations of thin-film tunnel junctions or point contacts with 1.018 V Weston saturated standard cells [22] calibrated by NBS. They obtained a value of 2*e*/*h* with a one-standard-deviation fractional uncertainty of 3.6 × 10^−6^. Finnegan et al. [39] reduced this uncertainty to 1.2 × 10^−7^ in 1971.

It was argued on fundamental grounds by Bloch [40] and Fulton [41] that Eq. (1) must be exact. The use of superconducting-quantum-interference device (SQUID) null detectors in the early 1970s allowed this to be tested to a few parts in 10^9^ [42, 43] and thus the Josephson effect had obvious potential for use as a voltage standard [44]. By the early 1970s, NIST staff had set up a potentiometric measurement system in Gaithersburg that compared 2 mV to 10 mV dc Josephson junction voltages with 1.018 V standard cells to within a few times 10^−8^ [45, 46]. International comparisons in 1971-72 between NMIs including NBS, the BIPM, the National Physical Laboratory (NPL) in the U.K., the National Research Council (NRC) in Canada, the National Measurement Laboratory (NML) in Australia, and the Physikalisch-Technische Bundesanstalt (PTB) in Germany found that the measured values of 2*e*/*h* agreed with each other to within 2 × 10^−7^ [47].

These results from the NMIs suggested the course of adopting a value of 2*e*/*h* for use in maintaining units of voltage. The U.S. was the first nation to do this, and the value of 2*e*/*h* to be used at NBS was chosen to prevent a discontinuity when NBS converted from standard cells to the Josephson effect [48]. NBS began maintaining and disseminating the U.S. volt based on the Josephson effect in July, 1972 using a 10 mV measurement system with an uncertainty of 2 × 10^−8^ [46]. Soon after, the Consultative Committee on Electricity of the CIPM recommended the value *K*_J−72_ = 483 594 GHz/V, which all countries adopted except the United States, France, and the Soviet Union.

In many applications, Josephson junctions were undoubtedly better references than standard cells, which are sensitive to environmental conditions, can shift values on transport, and can drift by a few times 10^−8^ per year. The typical 5 mV to 10 mV reference output from early Josephson devices made from a few junctions required both very low-level voltage balances and scaling by a factor of 100, both of which seriously limited the accuracy of measuring 1.018 V standard cells.

Then in 1977, Levinson [49] showed that unbiased Josephson junctions would spontaneously develop quantized dc voltages when irradiated with microwaves, opening the path to successful Josephson junction arrays. C. A. Hamilton, R. L. Kautz, F. L. Lloyd, and others of the NBS Electromagnetic Technology Division at Boulder began developing and improving Josephson standards based on series arrays of junctions operated near zero dc voltage bias [50, 51]. Elsewhere, Tsai et al. [52] found in 1983 that the constant of proportionality between the voltage and frequency is the same to at least 2 × 10^−16^ for two different kinds of Josephson junctions.

Stable 1 V zero-crossing arrays were operating at NBS [53] and PTB [54] by 1985, using about 1500 junctions and rf fields of 70 GHz to 90 GHz. Arrays with output voltages at the level of 1 V soon were used in NMIs throughout the world [55, 27]. By 1989 NIST had made a 19 000 junction, 12 V array [56]. The widespread use of Josephson junction arrays in national standards laboratories, and better SI determinations of 2*e*/*h*, led the CCE to recommend [57] a new exact conventional value for the Josephson constant:

$$K_{J-90}=483\;597.9\;\text{GHz/V},$$ (2)

which is fractionally larger by 8 × 10^−6^ than the 1972 conventional value. The new value was adopted worldwide on January 1, 1990, and thereby became the new basis for the U.S. Legal Volt. This definition of *K*_J−90_ is the present volt representation, based on an ideal Josephson voltage standard. The conventional value was assumed by the CCE to have a relative standard uncertainty of 0.4 μV/V. By convention, this uncertainty is not included in the uncertainties of the representation of the volt, since any offset from the SI volt will be consistent between different laboratories using the Josephson effect standard.

#### 1.4.2 The Quantum Hall Effect

In the classical Hall effect, a current of particles with charge *q* and velocity *v* is passed through a device placed in a magnetic field with perpendicular magnetic flux density component *B*. A Lorentz force *qvB* deflects the conducting charges toward one side of the device. The resulting charge redistribution produces an electric field *E* across the device. At equilibrium, a Coulomb repulsion force *qE* opposes the Lorentz force. The electric field generates a Hall voltage proportional to *B*, perpendicular to both the magnetic flux and the flow of current.

**Fig. 6:**
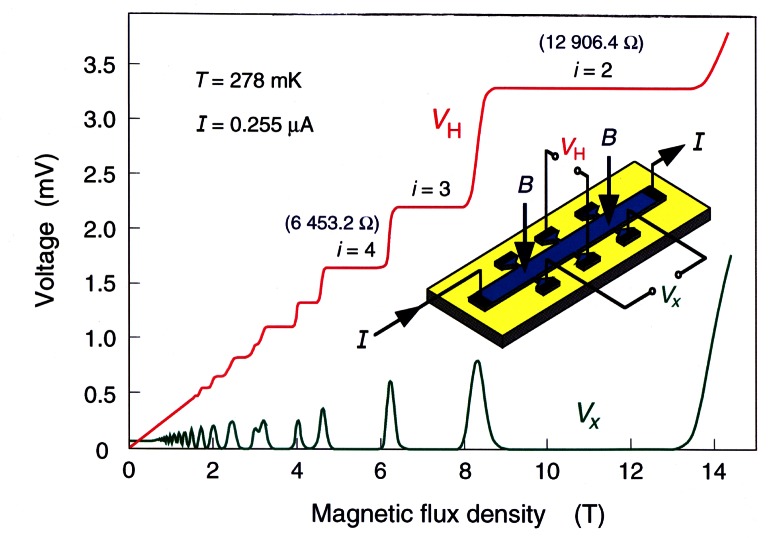
Graph showing the Hall voltage *V*_H_ and longitudinal voltage *V*_x_ for a quantized Hall resistance device in a magnetic flux density *B* and with constant current *I*. A diagram of such a device, pictured in the inset, shows the alignment of the flux density perpendicular to the plane of the device.

The QHE is seen only if the conducting particles (usually electrons) are confined to a two-dimensional sheet within the device by a potential that restricts their out-of-plane motion. In the integer QHE they can be treated as independent Fermi particles, and thus at low temperature form a two-dimensional electron gas (2DEG). This 2DEG is indicated by the light-blue region of the semiconductor device shown in the inset of Fig. 6. Once again, the Lorentz force resulting from the applied magnetic field equals the Coulomb force, generating a Hall voltage *V*_H_ across the device and a longitudinal voltage *V_x_* along the device; however, here the Hall voltage is no longer directly proportional to the magnetic flux density *B*.

Oscillations occur in the voltage *V_x_* of Fig. 6 when the applied current *I* is kept constant and the flux density *B* is varied. A series of constant-voltage plateaus arise in the *V*_H_ signal for those regions of *B* where *V_x_* is small. The transverse resistance, with a value of *R*_H_, is defined as the ratio of the quantum Hall voltage *V*_H_ across the device divided by the current *I* through the device, and thus has a constant value along a plateau. For example, two of the plateaus in the figure have measured QHR values of about 6 453.2 Ω and 12 906.4 Ω over a wide range of magnetic flux densities.

Klaus von Klitzing discovered the integer quantum Hall effect [58] during the night of February 4 and 5, 1980, at the High-Field Magnet Laboratory of the Max Planck Institute in Grenoble, France. He knew immediately that this discovery had significance for resistance metrology, because of his earlier experience as a summer student at the PTB. Von Klitzing contacted Volkmar Kose at the PTB to arrange a calibration of his reference resistors. The results, with a one-standard deviation uncertainty of about 10^−5^ *R*_H_, were announced at an international conference in June of 1980 and published [33] that August. He won the Nobel Prize in physics in 1985. Landwehr, his mentor, has written an account [59] of the events leading up to the discovery.

It is experimentally observed that the plateaus have the resistance values

$$R_{H}=h/e^{2}i,$$ (3)

where *i* is an integer and *h*/*e*^2^ is about 25 812.8 Ω. The *i* = 2, 3, and 4 plateaus are labeled in Fig. 6. Robert Laughlin, a co-winner of the 1998 Nobel Prize in physics for the development of an understanding of the fractional QHE, provides an elemental derivation [60] of Eq. (3).

This fundamental property arises because charges moving in crossed magnetic and electric fields obey the equation of motion, *qv_x_B_z_* = *qE_y_*, where *q* is the charge and *v_x_* is the velocity in the direction perpendicular to each field. *E_y_* can be written as the derivative of the electric potential *V_y_* with respect to the *y*-direction, which is the direction in which the Hall voltage is measured. Then,

$$\int_{V_{\min}}^{V_{\max}}{\text{dV}}_{y}=\int_{V_{\min}}^{V_{\max}}v_{x}B_{z}dy.$$ (4)

The integral on the left is just the Hall voltage, which is the Hall resistance divided by the total current. With the two-dimensional current density *J* = *ev_x_N*_S_ inserted on the right-hand side, the integration yields the equation *R*_H_ = *B*/*eN*_S_, where *N*_S_ is the number density of conducting electrons within the 2DEG. This equation is a classical result, which predicts a linear relation between resistance and the magnetic flux density *B*. In the QHE, near the centers of the plateaus, all the allowed states are filled and there is an energy gap to the next level. Equation (3) is found because of the following: (a) the electrons in the 2DEG make cyclotron orbits about the magnetic flux lines and occupy states in a Landau level; (b) the maximum number of states per unit area *n*_s_ in a filled Landau level is *n*_s_ = *eB*/*h*; and (c) *N*_S_ = *i n*_s_.

Workers in national metrology laboratories quickly began studying the effect. The uncertainty in measuring *R*_H_ needed to be reduced several orders of magnitude. By August, 1980, M. E. Cage, B. F. Field, and R. F. Dziuba from NBS were doing experiments [61] with R. Wagner at the Naval Research Laboratory in Washington, DC, initially using a Bitter magnet until a 13 T superconducting magnet was installed. Like von Klitzing, they used silicon metal-oxide-semiconductor field-effect transistors (Si MOSFETs) which have the unfortunate weakness that static electricity can puncture the oxide layer, rendering the device useless.

In 1982 D. Tsui and H. Stormer (1998 Nobel Prize co-winners with Laughlin for the fractional quantum Hall effect) invited NIST staff to Bell Labs in Murray Hill, NJ. Their GaAs/AlGaAs heterojunction devices, made by A. Gossard, were better than Si MOSFETs because of the smaller effective masses of the electrons in the 2DEG and wider plateaus. These devices, and improvements in the measurement system, enabled the first precision measurements of *R*_H_, with an uncertainty of 2 × 10^−7^ *R*_H_ [62].

Cage, Field, and Dziuba began monitoring the bank of five 1 Ω wire-wound resistors (whose average value was the U.S. Legal Ohm) at NBS in terms of the quantized Hall resistance *R*_H_ using an 8 T superconducting magnet supplied by Bell Labs. In 1983, Tsui, Stormer, and Gossard also gave NIST their best integer effect GaAs/AlGaAs devices. The potentiometric QHR measurement technique [63] and resistance scaling methods were refined, allowing NBS to monitor the U.S. Legal Ohm to within the fractional amount of 1.5 × 10^−8^ [29]. Over a period of 5 years leading up to 1988, measurements by Cage, C. T. Vandegrift, and Dziuba showed that the U.S. Legal Ohm was decreasing by a fractional amount of at least 5.3 × 10^−8^/year. The resistor-based legal ohms in other countries were also drifting with similar rates.

The relative uncertainties in the SI values of *h* and *e* were about 1 × 10^−6^ at that time, and so *h*/*e*^2^ could not be determined without absolute electrical standards linked to the SI quantities. Therefore, at NIST, the best SI value [28] of *R*_H_ was obtained using the calculable capacitor chain (described in Sec. 2.3). The resulting SI realization of *R*_H_ had an uncertainty of 2.4 × 10^−8^. The NPL in the United Kingdom [64] and the NML in Australia [65] also obtained SI values of *R*_H_, with relative standard uncertainties of 6.7 × 10^−8^ and 6.2 × 10^−8^, respectively.

The CCE considered these and other data in recommending the adoption of a new constant for maintaining the ohm [57]. The 1990 exact, conventional value of the von Klitzing constant,

$$R_{K-90}=25\;812.807\;\Omega ,$$ (5)

was adopted by all NMIs on January 1, 1990, and became the new basis for the U.S. Legal Ohm. The conventional value was assumed by the CCE to have a relative standard uncertainty of 0.2 μV/V. Again, by convention, this uncertainty is not included in the uncertainties of the representation of the ohm, since any offset from the SI ohm will be consistent between different laboratories using the QHE standard.

A set of technical guidelines [66] was also issued by the CCE to assure reliable measurements of *R*_H_ since it was found that the values can depend on parameters such as the device temperature [67], the applied current [68], and electrical contacts to the 2DEG [69]. It has been demonstrated, when following the technical guidelines, that the value of *R*_H_ is device-independent to a fractional amount of no more than 3.5 × 10^−10^ [70, 71].

#### 1.4.3 Ohm’s Law in Quantum Metrology

No deviation from the exact relations for the Josephson and quantum Hall effects [Eqs. (1) and (3)] has been proven either theoretically or experimentally, at least when the conditions approach the ideal. A third and complementary quantum physics effect is in development at a number of laboratories, based on single electron tunneling (SET). At NIST, these SET devices can be controlled so that an exact number of electrons can be generated (using tuned bias voltages) in a certain period of time for use in a measurement circuit. Proposals for comparing the Josephson voltage to that of a QHR device subjected to such an exact current [72, 73] are under study at NIST and other NMIs. Ohm’s law (*V* = *IR*) could then be applied directly to help determine the fundamental physical constants *e* and *h*.

It is, however, difficult to develop high enough currents for such experiments, because the SET current of a single device is limited by the capacitance and the time constant of the device to about 1 pA [74]. In addition, “charge offsets” [75] prevent large numbers of devices from working simultaneously in the same circuit (due to complexity in tuning the circuit), and it has not yet proved possible to raise the current by running many of the devices in parallel. This special limitation on current so far has prevented any significant measurements using SET currents in QHR devices. Instead, SET research has led M. W. Keller, A. L. Eichenberger, J. M. Martinis, and N. M. Zimmerman at NIST to pursue a cryogenic capacitance standard [76] that is charged by a SET device, for which only a small current is required.

## 2. Some Present-Day Electrical Measurement Programs at NIST

### 2.1 DC Voltage

The volt is a derived unit in the SI, one definition of which is the potential difference between two points on a conductor carrying a current of one ampere when the power dissipated is one watt. NIST maintains the representation of the volt based on the Josephson effect, described in Sec. 1.4 as a simple relationship between the voltage across a superconductor—insulator—superconductor junction and the microwave frequency radiating onto the junction. The relationship can be expressed by the equation *V* = *nf*/*K*_J_, where *V* is the quantized voltage, *n* is an integer, *f* is the microwave frequency, and *K*_J_ = 2*e*/*h* is the Josephson constant determined by the Plank constant *h* and the elementary charge *e*.

#### 2.1.1 The NIST DC Voltage Standard Laboratory

**Fig. 7:**
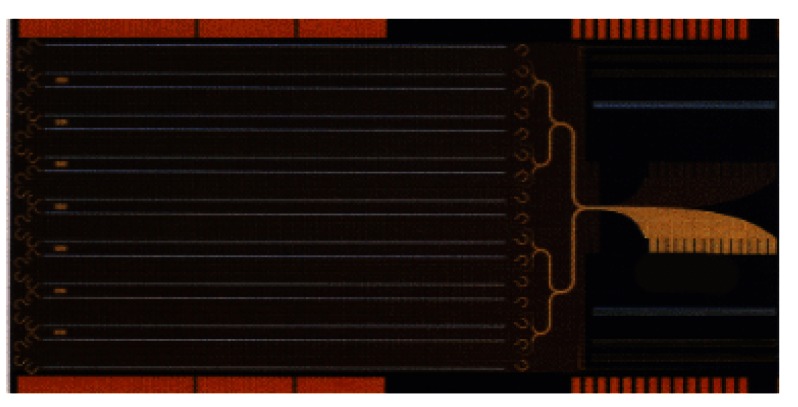
This picture shows a Josephson junction array (18 mm × 9 mm) consisting of 20 208 junctions in series that is able to provide voltages up to 12 V with ≈ 155 μV between two adjacent steps, when irradiated with microwave radiation at a frequency of 75 GHz. Each Josephson junction provides zero-crossing voltage steps when microwave radiation is applied through the finline antenna at the right.

**Fig. 8:**
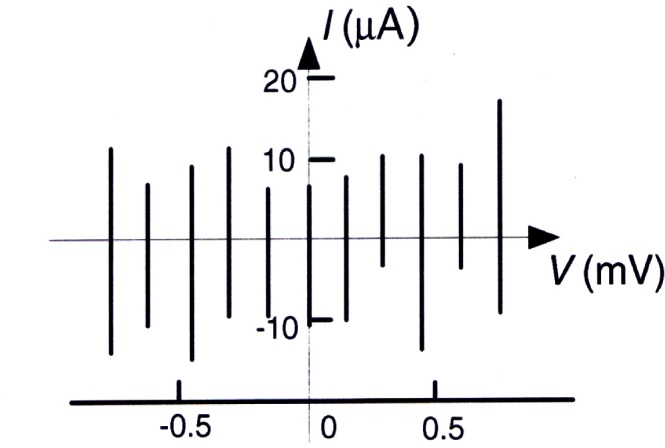
Graph of zero-crossing voltage steps for a single Josephson junction.

The responsibilities for maintaining and disseminating the volt through NIST calibration services fall to the Electricity Division’s Josephson voltage and voltage-calibration laboratories. Two Josephson voltage standard (JVS) systems, designated NIST-1 V and NIST-10 V, operate as the U.S. representation of the SI volt. The NIST-1 V system uses a 1 V Josephson array consisting of 2076 junctions in series. The NIST-10 V system uses a 10 V array consisting of 20 208 junctions. Figure 7 shows a picture of a 10 V Josephson array made of niobium and Fig. 8 shows the zero-crossing steps that each junction of the array can provide.

These two Josephson voltage standard systems are compared every year by measuring a set of Zener voltage standards to check the correctness of JVS operation. The difference between the two JVS measurements at 1.018 V in the last two comparisons has been less than 1 nV, with a combined expanded uncertainty of less than 6 ×10^−9^ (coverage factor *k* = 2) [77]. The NIST-1 V system is dedicated to transfer the NIST representation of the SI volt to a primary group of standard cells on a regular basis. The NIST-10 V system is used for evaluating new voltage standards and system software, to develop a voltage measurement assurance program and to participate in international and domestic JVS intercomparisons.

A comparison between a portable BIPM JVS and the NIST-1 V JVS at 1 V was carried out in 1992 with a relative agreement of 1.3 × 10^−8^ (*k* = 2) for an indirect comparison (via mutual measurements of an isolated Zener voltage standard) and 3 × 10^−10^ (*k* = 2) for a direct comparison [78]. A recent comparison between the BIPM and both NIST sites (Gaithersburg and Boulder) involved measuring three transport Zener standards. The fractional differences among the 10 V calibrations traceable to these three JVS lie within 2.6 × 10^−8^ with a combined expanded uncertainty of 3.4 × 10^−8^ (*k* = 2) [79].

**Fig. 9:**
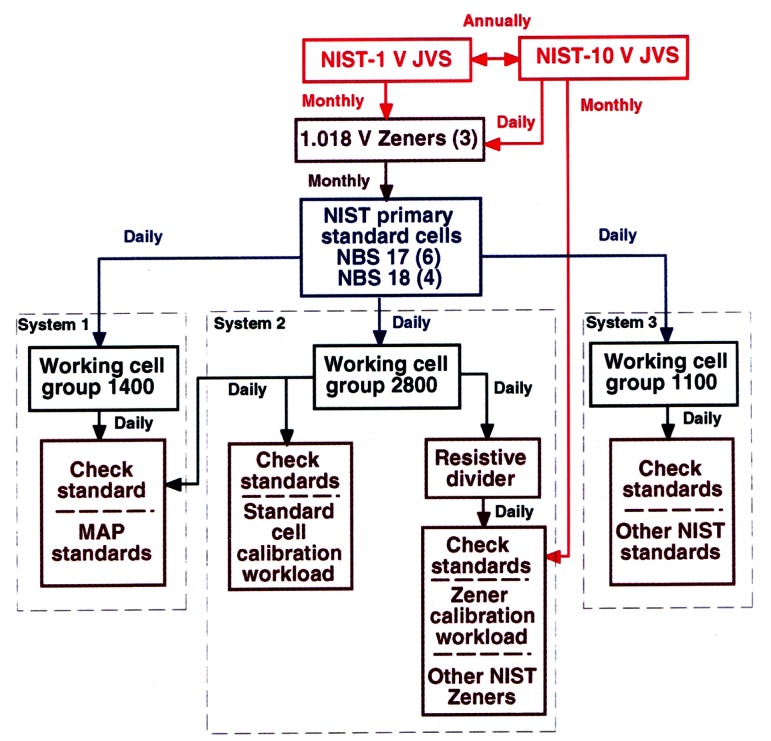
Voltage calibration path at NIST, showing the Josephson voltage standard (JVS) 1 V system and 10 V system. Also shown are the transfer Zener voltage references, primary standard cells, and three calibration systems. MAP standards are standard cells used for the measurement assurance program.

Figure 9 describes the traceability of the dc voltage calibration service to the SI. The NIST voltage calibration laboratory maintains a primary group of ten Weston cells, housed in two separate enclosures with a temperature stability of 10 μK. Because of the risk that a standard cell could be damaged by a sudden change in the voltage of the JVS, the cells are not calibrated directly against the JVS. Instead, the primary group of standard cells is calibrated through a set of three Zener transfer standards measured daily against NIST-10 V and monthly against NIST-1 V, and is maintained with a relative standard uncertainty of 3 × 10^−8^.

There are three measurement systems that perform voltage calibrations, as shown in Fig. 9. Each of the systems has a working cell group, calibrated daily against the primary group of standard cells. All of the NIST standard cell groups have better long-term predictability and lower medium and short-term noise compared to Zener standards. The main external calibration workload, containing customer standard cells and Zener standards, is measured against working cell group 2800. As a check, a Zener voltage standard is calibrated daily against the working cell group 2800 through a resistive ratio divider. This Zener standard is also calibrated monthly against NIST-10 V to check the consistency of the Zener calibrations, which are made through a resistive ratio divider.

#### 2.1.2 Industrial Needs in Voltage Metrology

**Fig. 10:**
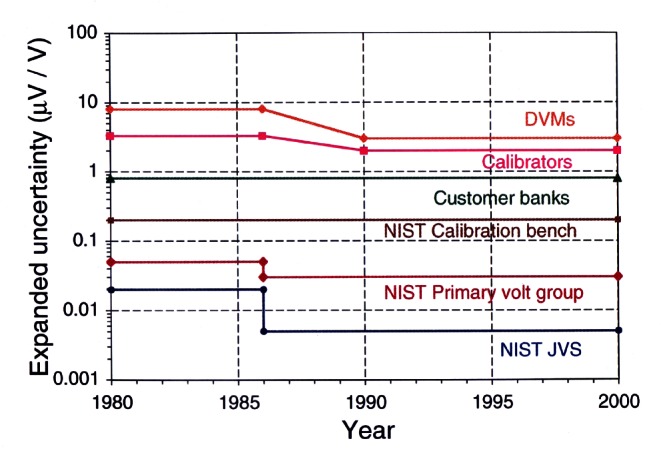
Industry needs and NIST capabilities in voltage calibration. The expanded uncertainty corresponds to a coverage factor *k* = 2 (DVM: digital multimeter, JVS: Josephson voltage standard).

Figure 10 shows the progression in NIST’s capability in voltage metrology, compared with industry needs. In the early 1960s, voltage measurements using a voltmeter required a relative uncertainty of several hundred times 10^−6^. Today, a high-end digital voltmeter is able to make voltage measurements with a relative uncertainty of three or four times 10^−6^. Meeting the needs of instrument manufacturers, calibration laboratories, and military laboratories requires a NIST voltage calibration capability with a relative uncertainty of a few times 10^−7^. The NIST dc voltage calibration service described here also supports NIST calibration services for high-precision digital multimeters and calibrators. The typical turnaround time is about 3 weeks for Zener standards. The time required can be several months for saturated standard cells; this depends on how quickly the standards reach equilibrium after transport.

The term “intrinsic standard” is sometimes used to describe a type of standard, such as a JVS, QHR standard, triple point cell, deadweight pressure gauge, etc., based on physical laws rather than on the stability of physical artifacts which depend on bulk materials properties. There are approximately twenty industrial and military calibration laboratories throughout the United States that operate a JVS as a basis for traceable calibration measurements. The JVS consists of many cryogenic and microwave components, and each of these, as well as the environment and user technique, can contribute uncertainty to the voltage measurement. Accordingly, it is necessary to make intercomparisons among independent JVS laboratories, to ensure the correctness of the measurements in these laboratories, just as it is at the international level. In 1991 NIST conducted the very first JVS laboratory comparison experiment using transportable 10 V Zener standards, in which five other U.S. industrial and military laboratories participated [80]. Such comparisons are now carried out regularly under the auspices of the National Conference of Standards Laboratories, an industry trade association, with support from NIST as necessary.

Most of the intercomparisons between Josephson voltage standards use such a set of transport Zener standards. In most cases, the noise characteristics of the Zener standards are the limiting factor in the uncertainty. Moreover, Zener standards are subject to changes due to environmental conditions of temperature, barometric pressure, and relative humidity. The uncertainty of an intercomparison can be significantly improved if corrections for the effects of these environmental conditions are made. NIST is now developing a measurement assurance program (MAP) [81] using a set of low noise and well-characterized Zener standards to improve the uncertainty of a MAP for JVS intercomparisons. It is foreseeable that a relative standard uncertainty of 5 × 10^−8^ (*k* = 2) or better for a MAP at 10(V level can be achieved using this approach.

The challenge NIST is facing in the new millennium is to meet even greater needs in industrial applications and scientific research for more reliable, accurate and economical voltage sources and for measurement techniques. For example, a project to improve the present JVS intercomparisons is currently under way. This entails development of a compact JVS system that can be shipped to another lab for a direct or indirect comparison with a second JVS. The compact JVS will enhance the capability of the NIST calibration service for customers who base their measurements on a JVS by offering them direct traceability at an uncertainty level of a few parts in 10^9^.

#### 2.1.3 Future Developments in Voltage Metrology

When the CCE recommended international acceptance of the value of the Josephson constant *K*_J−90_ = 483 597.9 GHz/V beginning Jan. 1, 1990, they believed this provided a volt based on the Josephson effect that agreed with the SI volt with a relative standard uncertainty of 4 × 10^−7^. A recent moving-coil watt balance experiment carried out at NIST, based on equating electric power and mechanical power, has determined the Josephson constant to be 483 597.892 GHz/V, with a relative standard uncertainty of 4.4 × 10^−8^ [82]. The agreement between the Josephson constant determined in terms of the SI and the value of *K*_J−90_ has thus been improved by an order of magnitude. This result suggests that the value of *K*_J−90_ was well defined, and that there is no need to make an adjustment of the Josephson constant in the near future.

The progress in technology over the last thirty years has made it possible to integrate tens of thousands of Josephson junctions on a single chip and to generate voltages up to 10 V. Now, there are active research and development activities to manufacture more economical and reliable Josephson voltage standards for better voltage measurements. Results in high temperature superconductor research may create a new family of Josephson junctions usable at liquid nitrogen temperature (77 K) or at even higher temperatures. Progress in cryogenics has encouraged manufacturers to develop efficient cryo-coolers for cooling a conventional Josephson array to its working temperature (4 K to 5.2 K), saving the cost of liquid helium.

C. J. Burroughs, S. P. Benz, C. A. Hamilton, and T. E. Harvey, working at the NIST Boulder laboratories, have developed a new type of array with programmable binary segments of Josephson junctions. The programmable array has step-amplitude a hundred times larger than the conventional zero crossing steps [83]. To improve the NIST voltage standard laboratory, such a programmable array working at the 1 V level will be tested as a backup to the NIST primary group of standard cells. This array might eventually replace the primary group of standard cells, thereby reducing the system uncertainty by shortening the steps in the voltage dissemination chain. The noise immunity and high resolution in voltage measurements provided by the programmable array now allows its use in the NIST watt balance experiment, thereby reducing the uncertainty of the voltage reference by a factor of eight. In the future, an ultimate Ohm’s law or “quantum triangle” experiment (combining the Josephson effect, the quantum Hall effect, and the single-electron-tunneling device) may be based on this type of JVS.

### 2.2 AC-DC Thermal Transfer Instruments

#### 2.2.1 Development of the Thermal Converter at NIST

AC voltages and currents in the frequency range from low audio to hundreds of megahertz are measured at the best uncertainties by comparison to dc standards with ac-dc thermal transfer instruments. The field of ac-dc thermal transfer metrology was essentially launched by the publication of a paper nearly half a century ago (1952) in the *Journal of Research of the National Bureau of Standards*, “Thermal Converters as AC-DC Transfer Standards for Current and Voltage Measurements” [84]. This paper by F. L. Hermach laid the foundation for the techniques of ac-dc transfer and provided the theoretical basis for the ac-dc thermal transfer structures. These devices were first used for accurate calibration of ammeters and voltmeters in the audio frequency range [84] and later at radio frequencies [85] for measurements of voltage, current, and power. Hermach and the staff of the NBS Electricity Division produced important developments including the first transmission-line analysis of coaxial transfer standards [85].

In general, the rate of transformation of energy from electrical to thermal form in thermal converters is proportional to the root-mean-square (rms) values of current and voltage. The heater temperature is a function of the square of the heater current even if the constants in the defining equation, covering the underlying physics, vary with temperature or time. Since the response of thermal converters is calibrated on direct current at the time of use, ac-dc transfers are possible with little decrease in accuracy from drift or external temperature influences.

Traditional thermal converters contain wire heaters or thin, metal heater structures. The temperature of the heater is typically monitored with one or more thermocouples, also made of wire or thin metal film. The best-performing primary standards usually contain many thermocouples in an arrangement that minimizes ac-dc difference by reducing both heater temperature and thermal gradients. Current research at NIST includes two areas directed at new thermal converters suitable for both primary and working standards.

#### 2.2.2 Thin-film Multijunction Thermal Converters

Multijunction thermal converters (MJTCs) are used in very high-accuracy ac-dc difference metrology because they have very small ac-dc differences, follow the rms law of excitation, and produce high output emfs. MJTCs traditionally have been fabricated from wire heater resistors and thermocouples. The project to develop thin-film MJTCs (FMJTCs) involves the use of micro-machining of silicon and photo-lithography on thin films to produce high-performance thermal transfer standards. Multi-layer FMJTCs have been designed, fabricated, and tested at NIST by J. R. Kinard, D. B. Novotny, and D. X. Huang, and new improved converters are under development [87].

**Fig. 11:**
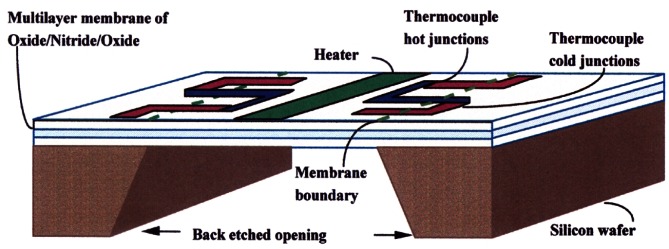
Cross section of a thin-film multijunction thermal converter.

The basic elements of the devices are a thin-film heater on a thin dielectric membrane, a silicon frame surrounding and supporting the structure, and thin-film thermocouples positioned with hot junctions near the heater and cold junctions over the silicon. Carefully selected materials in new thermal designs are required, along with very accurate dimensioning of the heater and thermocouples. The heater and thermocouples are sputter deposited and patterned with photolithography. Contributions to ac-dc difference from the Thomson effect and other effects are further reduced by the appropriate choice of heater alloy. Figure 11 shows a cross section of an FMJTC.

**Fig. 12:**
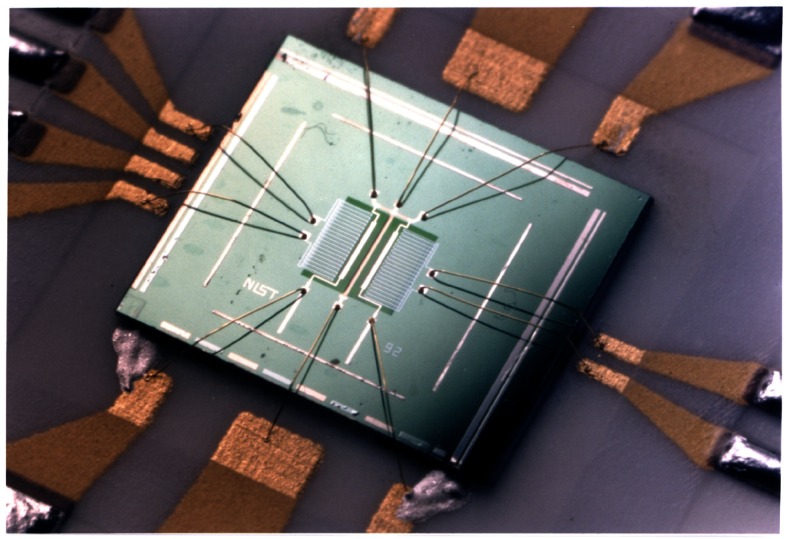
Integrated micropotentiometer including the thin-film multijunction thermal converter (FMJTC) structure.

Integrated micropotentiometers are thermal transfer devices that contain FMJTCs and thin-film output resistors fabricated as an integrated structure on the same silicon chip. Figure 12 shows an integrated micropotentiometer including the FMJTC structure. New versions of the FMJTCs and integrated micropotentiometers are under development that include new membrane materials and vacuum packaging, with the help of novel etching techniques such as front and back surface etching.

#### 2.2.3 Cryogenic Thermal Converter

At audio frequency, thermal and thermoelectric effects ultimately limit the measurement uncertainty in conventional room-temperature thermal converters. Heater powers as high as a few tens of milliwatts and temperature differences as high as 100 K are common in some thermal converters. To reduce these effects and to achieve very high temperature sensitivity, a novel sensor employing a superconducting resistive-transition edge thermometer is being developed at NIST by C. D. Reintsema, E. N. Grossman, J. A. Koch, J. R. Kinard, and T. E. Lipe [88, 89]. Since the new converter operates at temperatures below 10 K and is mounted on a platform with precise temperature control and very small temperature gradients, the thermal and thermoelectric errors are potentially quite small. Because of the very high temperature sensitivity of the superconducting transition, this converter also offers the possibility of direct thermal transfer measurements at very low signal levels.

**Fig. 13:**
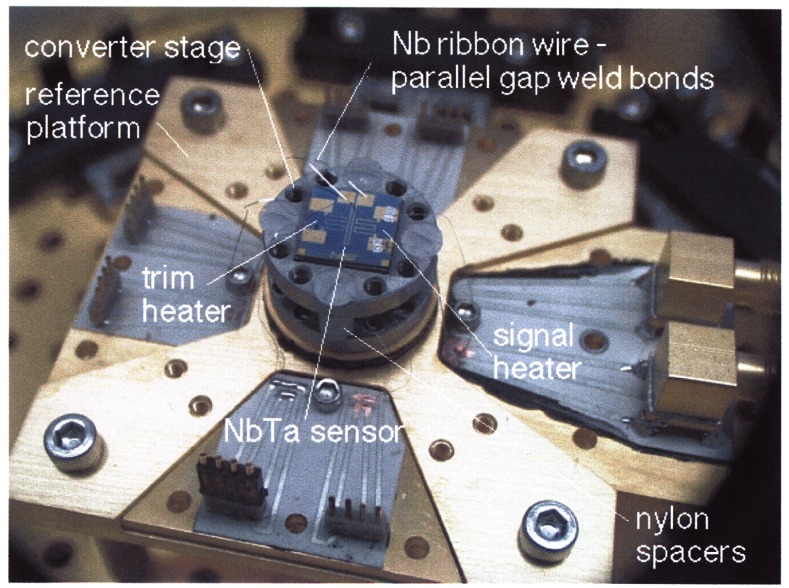
Photograph of a cryogenic thermal transfer standard showing the chip, converter stage, and reference platform. The cryostat base-plate is at 4.2 K, the reference platform is at 6 K, and the converter stage is at 6 K + Δ*T*_c_.

Figure 13 shows the experimental platform for the prototype cryogenic thermal converter. This transfer standard consists of a signal heater, trim heater, and temperature sensor all mounted on a temperature-stabilized platform. The sensor resistance is measured by an ac resistance bridge, and the temperature of the assembly is held constant by the closed loop application of power to the trim heater. A NbTa thin-film meander line is used as the thermal sensor and is thermally biased to operate within its superconducting-resistive transition region. The signal heater in the prototype device is a 7 Ω thin-film meander line and the trim heater is a 450 Ω PdAu thin-film meander line, both adjacent to the detector on the silicon substrate. To ensure temperature stability, the entire converter assembly is mounted on a second platform controlled at a slightly lower temperature. This intermediate stage is thermally isolated and is controlled by a second ac resistance bridge using another transition edge sensor and heater.

Using this new cryogenic converter, measurements have been made at signal power levels of a microwatt, which is around 1000 times lower than is possible with room-temperature converters. Characterization using a fast-reversed-dc source has shown that the thermoelectric errors are presently in the 1 μV/V to 2 μV/V range. These early results are encouraging, but considerable improvement both in the resistance bridge performance and in the input transmission line will be necessary for this new device to be a candidate for consideration as a primary standard.

### 2.3 Impedance Measurements

#### 2.3.1 Development of the Calculable Capacitor at NIST

The link between the QHR standard and SI units was derived from modern experiments, including primarily the NIST calculable capacitor and the calculable capacitor chain. The calculable capacitor experiment is similar, in the degree of importance in modern electrical metrology, to the Josephson and quantum Hall effects. However, only a few NMIs maintain calculable capacitor experiments, while dozens now maintain JVS and QHR measurement systems.

Thompson and Lampard [90] first published the theory behind the calculable capacitor in 1956. The equation below shows that the two cross capacitances *C*_1_ and *C*_2_ for a specially designed cylindrical cross capacitor can be found if the length of the electrodes *L* is known (ε_0_ is the electric constant):

$$\exp\left(-\pi \frac{C_{1}}{L\varepsilon_{0}}\right)+\exp\left(-\pi \frac{C_{2}}{L\varepsilon_{0}}\right)=1$$ (6)

The first report of a measurement based on a calculable cross-capacitor at NIST was in 1961 by Cutkosky [13]. In addition to measurements of the NIST primary standard of capacitance, this effort entailed the use of a special quadrature bridge by which a resistor with a known frequency response could be measured, first relative to a capacitor, and then used to measure resistance standards. In this fashion, Cutkosky obtained the value of the U.S. Legal Ohm with a relative standard uncertainty of 2.1 × 10^−6^ using horizontally-mounted cylindrical gauge bars as capacitor electrodes. Around the same time, Clothier at the National Measurement Laboratory in Australia constructed a calculable capacitor consisting of four vertical electrodes [91]. This was used, starting in 1963, in Australian experiments to realize the (SI) farad and (SI) ohm with a relative standard uncertainty of less than 1 × 10^−7^.

After the success of Clothier’s calculable capacitor design, a new cross-capacitor was constructed at NIST in the late 1960s utilizing a similar geometry. This capacitor is the one used at NIST today, although with several improvements. In 1974, Cutkosky reported the first SI value of the U.S. Legal Farad and the U.S. Legal Ohm [92] derived from this calculable capacitor. At the time, the national units of impedance were defined as the average value of a bank of 10 pF fused silica capacitors [93] in the case of capacitance and a bank of 1 Ω Thomas-type resistors for resistance. The realization of the ohm from the calculable capacitor at NBS in terms of the SI units of length and time was evaluated to have a relative standard uncertainty of 0.03 × 10^−6^. Shields et al. [28] reported the second NIST realization of the ohm and farad in 1989 using the same system after making several improvements. The relative uncertainties were 0.022 × 10^−6^ for the U.S. Legal Ohm and 0.014 × 10^−6^ for the U.S. Legal Farad. Jeffery et al. reported [94] the most recent realization of the ohm in 1996 with a relative uncertainty of 0.024 × 10^−6^.

#### 2.3.2 The NIST Calculable Capacitor

**Fig. 14:**
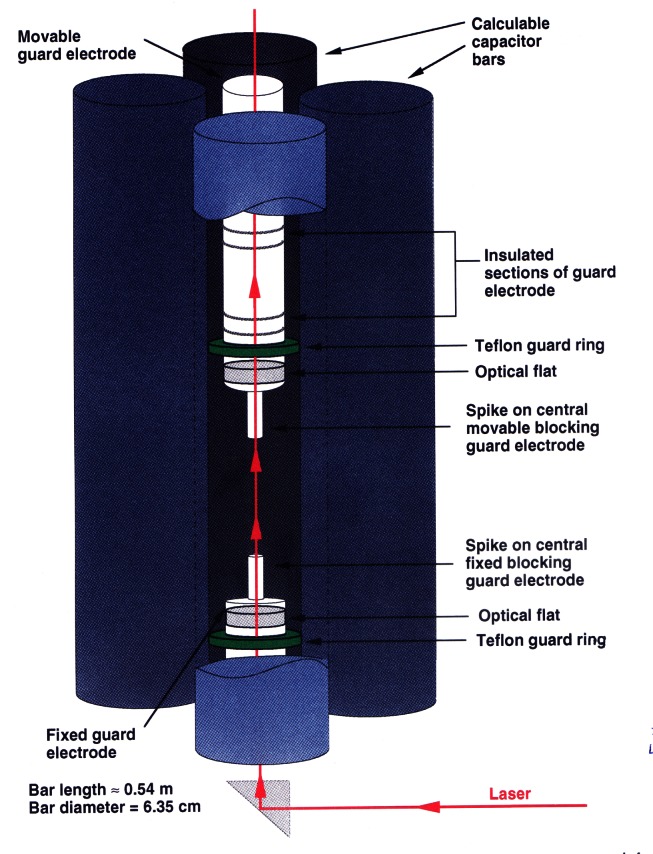
Diagram of the calculable capacitor electrodes, with one of the four main electrodes shown in cut-away view. Measurement voltages are applied across opposite pairs of main electrodes, and the central blocking electrode can be moved vertically to change the capacitance.

The calculable capacitor consists of four vertical cylindrical bars arranged at the corners of a square in the *X*-*Y* plane, placed symmetrically about the central *Z*-axis (see Fig. 14). Capacitance is measured between diagonal pairs of opposite bars, which for this geometry gives two capacitance values *C*_1_*'* ≈ *C*_2_*'* (each per unit of length). The Thompson-Lampard equation [Eq. (6)] reduces to *C'* ≈ 2 pF/m, where

$$C^{\prime }=\left(C_{1}^{\prime }+C_{2}^{\prime }\right)/2.$$ (7)

Second-order terms due to the difference between C_1_*'* and *C*_2_*'* contribute less than 10^−9^ *C'* to this capacitance in the NIST calculable capacitor.

The working length of the calculable capacitor is defined by two cylindrical electrodes on the central *Z*-axis. Except in the space between the ends of these electrodes, the electric field between opposite capacitor bars is completely blocked. A change in the vertical position of either of these grounded electrodes effectively changes the length associated with *C*_1_*'* and *C*_2_*'*. In practice, the lower blocking electrode is fixed and the upper one is moved to adjust the capacitor’s value. Measurements are made by comparing a fixed-value 10 pF capacitor to the calculable capacitor at two positions of the moveable electrode, where the values are 0.2 pF and 0.7 pF. Displacement of the electrode between these two positions yields a difference of 0.5 pF. By measuring the displacement of the blocking electrode rather than the absolute length of the capacitor, many problems associated with fringing effects at the ends of the capacitor are eliminated.

**Fig. 15:**
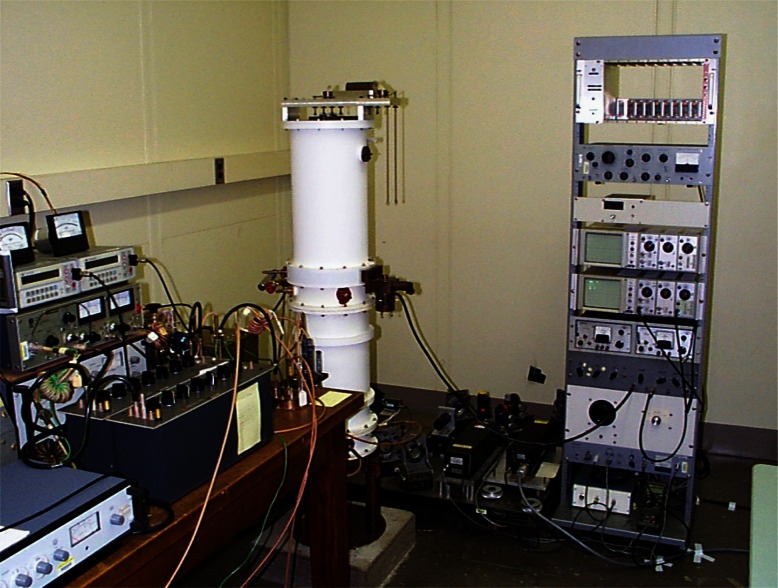
Calculable capacitor (vacuum chamber at center) and measurement apparatus.

A Fabry-Perot interferometer measures the relative displacement of optical flats mounted in the moveable and fixed blocking electrodes. The Fabry-Perot interferometer employs a fringe-locking laser optical system. The system is enclosed in a metal case, which is kept under vacuum. This eliminates the need to apply corrections due to the dielectric constant of air and provides a clean environment for the electrodes. The calculable capacitor apparatus is shown in Fig. 15. From the Fabry-Perot length measurement and Eq. (6), the value of the calculable capacitor is determined and a value is assigned to the fixed capacitor through comparison in an ac capacitance bridge [92].

The present relative combined standard uncertainty for this measurement [95] is 0.019 × 10^−6^ and the sources of this uncertainty are listed in Table 1. The largest relative standard uncertainty in Table 1 is that due to geometric imperfections. This includes the relative alignment of the axes of the bars to each other and to the blocking electrodes, alignment of the electrical axis of the capacitor and the optical axis of the interferometer, and imperfections in the bars. The magnitude of the uncertainty attributed to geometrical imperfections is the standard deviation of measurements of 0.1 pF increments made along the length of the capacitor. The blocking electrode end is presently a cylindrical spike, but recent tests by Jeffery [96] have shown that a modified cone shape (cone with a very short cylindrical spike) could reduce the effect of geometric imperfections in the bars.

#### 2.3.3 Realization of the SI Ohm: The Calculable Capacitor Chain

**Fig. 16:**
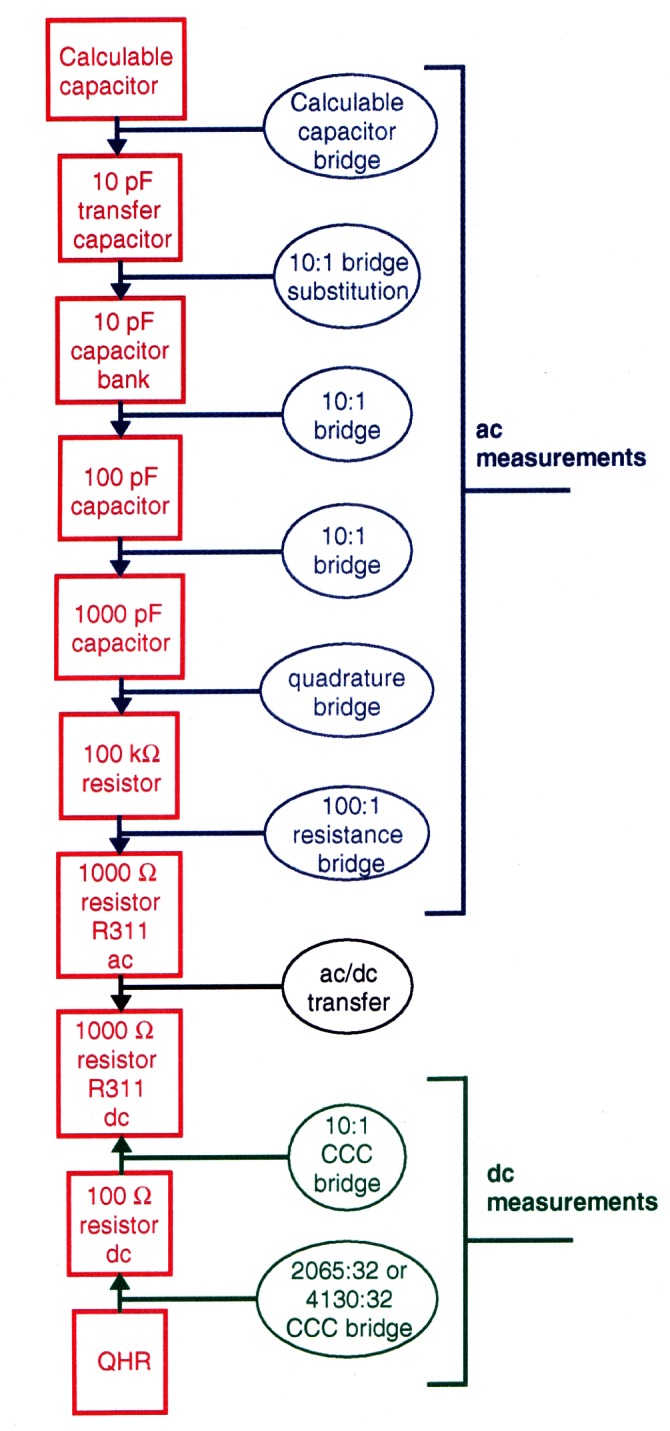
The calculable capacitor measurement chain. Capacitance and resistance standards are represented by red boxes, with QHR representing the quantized Hall resistance. Measurement bridges used to relate the standards to each other are represented by ovals.

Since 1990, the U.S. Legal Ohm has been defined by the internationally agreed upon value of the *i* = 1 resistance plateau of the QHR, *R*_K−90_. The link between the calculable capacitor and the ohm is made through a sequence of measurements called the calculable capacitor chain. This sequence is shown in Fig. 16. Measurement of the QHR via the calculable capacitor provides one of the best determinations, expressed in terms of SI units, of the von Klitzing constant *R*_K_ and the fine-structure constant *α* [57, 97, 98]. The inverse fine-structure constant *α*^1^ can be obtained from the measured value of *R*_K_ and Eq. (6) with no additional uncertainty since it is generally accepted that *R*_K_ is related to the fine-structure constant *α* by

$$R_{K}=h/e^{2}=\mu_{0}c/2\alpha ,$$ (8)

where *h* is the Planck constant and *e* is the elementary charge. The magnetic constant (permeability of vacuum), μ_0_ = 4π × 10^−7^ N/A^2^, and the speed of light in vacuum, *c* = 299 792 458 m/s, are exactly defined in the SI.

The 1989 NIST value of the von Klitzing constant is *R*_K_ = 25 812.807 23(61) Ω, and the 1996 value is *R*_K_ = 25 812.808 31(62) Ω, which is larger by a fractional amount of 4.2 × 10^−8^. The difference in the two results is small but significant, and we believe that the most recent result is more reliable as it is based on a series of measurements and not on one measurement as was the 1989 result. From our most recent determination, we find that *α*^−1^ = 137.036 003 7(33). Both of the above are in close agreement with recent quantum electrodynamic (QED) calculations of the anomalous magnetic moment of the electron *a*_e_ by Kinoshita [99], which may be combined with an accurate experimental value of *a*_e_ to derive *a*. This combined experimental-theoretical assignment, *a^−^*^1^ = 137.035 999 58(52) is 3.0 × 10^−8^ smaller than the 1996 value NIST reported.

#### 2.3.4 Maintenance of the Capacitance Unit from the Calculable Capacitor

Including all the preliminary calibration measurements, the calculable capacitor realization of the farad requires on the order of 1 month to complete. A 10 pF reference capacitor, measured against the calculable capacitor, is taken to another laboratory and the unit transferred to a 10 pF reference bank of four fused silica capacitors, which are the representation of the unit between calculable capacitor measurements.

**Fig. 17:**
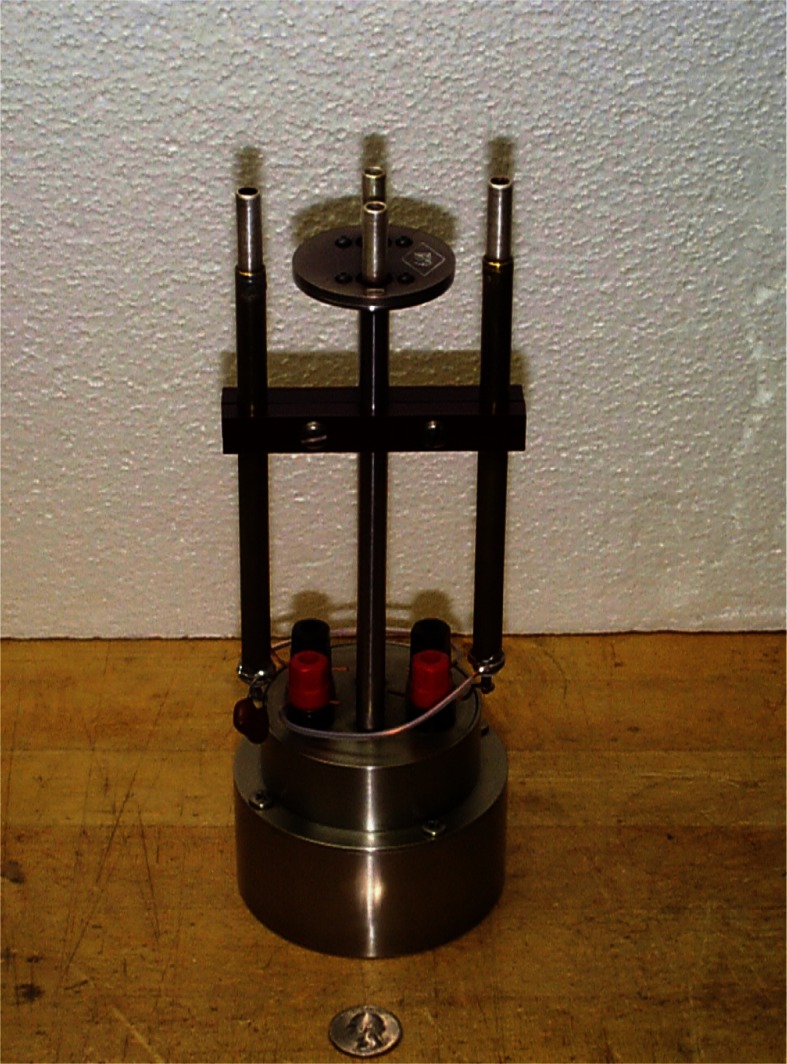
A 10 pF fused-silica capacitance standard.

The NIST 10 pF capacitor reference bank capacitors are intercompared weekly, along with several other capacitors of the same type, to monitor their behavior over time. The 10 pF fused silica capacitors were developed at NIST by R. D. Cutkosky and L. H. Lee [93], and have a very low average fractional drift rate of 2 × 10^−8^ per year. One of the 10 pF fused silica capacitors is shown in Fig. 17. These capacitors are sensitive to dimensional changes, and have a temperature coefficient of 10 μF/F per °C. Thus, they are kept in a temperature-controlled oil bath at (25 ± 0.0001) °C. Inside each standard, a calibrated copper resistor in close thermal contact with the capacitance element allows the temperature of the capacitor to be recorded when the capacitor’s value is measured in order to correct the capacitance value to a selected reference temperature.

#### 2.3.5 Impedance Calibration Laboratory

The NIST impedance calibration laboratory (ICL) disseminates the SI units (the farad and henry) through capacitance and inductance calibrations for customers, both inside and outside of NIST. The ICL provides calibrations of nominal-valued capacitors in the range from 0.001 pF to 1 μF in the frequency range from 100 Hz to 10 kHz. Customers include aerospace companies, instrumentation companies, the U.S. armed forces, secondary calibration laboratories, and other U.S. and foreign national laboratories. The laboratory also provides capacitance calibrations at 1 kHz that are used by the high-frequency calibration laboratories at NIST for their calibrations at frequencies above 1 MHz. The ICL provides calibrations of inductors in the range 50 mH to 10 H in the frequency range from 65 Hz to 10 kHz. The inductance value is found using the Maxwell-Wien bridge [100], which derives the value of the inductor by comparison against two resistors and a capacitor.

#### 2.3.6 Extension of Measurement Frequency

One of the main areas of research in impedance is the extension of capacitance measurements to other frequencies besides 1592 Hz (*ω* = 10^4^), the designed operating frequency of the calculable capacitor. This frequency was chosen to allow the calculable capacitor chain to transfer the impedance value of a 1000 pF capacitor to that of a 100 kΩ resistor.

Presently the primary laboratory operates only at 1592 Hz. The capacitance unit is transferred from the calculable capacitor to the ICL via fused silica dielectric capacitors whose value has been determined only at 1592 Hz. However, the ICL performs customer calibrations at 10 kHz, 1000 Hz, 400 Hz, and 100 Hz and the frequency dependence of the fused silica capacitors has a relatively large uncertainty. NIST plans to develop multi-frequency measurement capabilities to better support our customers needs in the frequency range from 100 Hz to 10 kHz. This will require design and construction of capacitance bridges that work at multiple frequencies as well as an evaluation of the calculable capacitor system at these frequencies.

#### 2.3.7 AC QHR Measurements

The calculable capacitor is one of the most direct ways to obtain the SI farad and allows the realization of the SI ohm. However, it is a very difficult and resource intensive experiment; thus only a few NMIs in the world have implemented it. Typical set-up times are from five to ten years including precision machining and detailed evaluation of the system. Furthermore, the present calculable capacitor uncertainty is close to its expected limit due to the macroscopic nature of the experiment. There has been much interest in finding a way to obtain a capacitance unit by other means. An ac determination of impedance based on the QHR is one of these alternatives.

Since most NMIs already have a dc QHR system for resistance calibrations, it would be very convenient if the quantum Hall effect could be used for impedance determinations as well. This would be a reversal of the chain of measurements that is used to realize the SI ohm without the ac/dc conversion step (See Fig. 16). If the QHR could be used directly with a quadrature bridge, which relates capacitance to resistance, this chain could be made even shorter. Several national laboratories [101, 102, 103, 104, 105] have begun working on ac QHR measurements with the hope that the ac QHR could provide a working unit similar to the internationally agreed upon value of the dc QHR.

Several difficulties have been encountered with the ac QHR measurements, so far producing a much higher limit on the uncertainty than with the dc QHR. These include an observed linear frequency dependence and differences from the dc value of about 1 × 10^−7^. NIST has begun to develop an ac QHR measurement system. As one of the few laboratories in the world able to link the dc QHR to the calculable capacitor, NIST will be able to compare the ac and dc QHR measurement chains as well as determine SI values of the ac QHR.

### 2.4 DC Resistance

#### 2.4.1 Quantized Hall Resistance Measurements

Shortly after the discovery of the QHE, NBS developed a system based on the QHE to monitor the U.S. Legal Ohm, maintained by five Thomas-type resistance standards, with a relative uncertainty of a few times 10^−8^ [106]. This consisted of a constant current source, a potentiometer, and an electronic detector. The current source energized the QHE device and a series-connected reference resistor of nominal value equal to the QHR. With the potentiometer balancing out the nominal voltage across either resistance, the detector measured the small voltage difference between the QHE device and reference resistor. Scaling down to the 1 Ω level was accomplished using specially-constructed Hamon transfer standards.

**Fig. 18:**
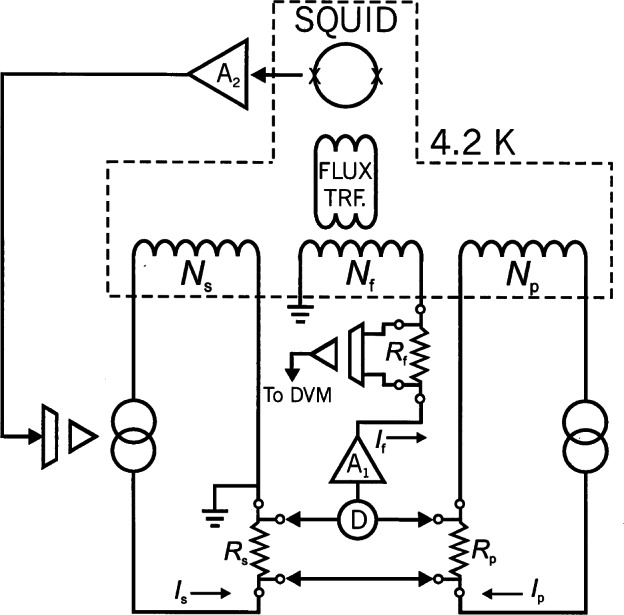
Schematic of a cryogenic current comparator resistance bridge.

Starting in 1991, a QHR laboratory was set up near the resistance calibration laboratory for the routine maintenance of the U.S. legal ohm. This laboratory uses a cryogenic current comparator (CCC) resistance bridge [107], which in a two-step process can compare the QHR to a 1 Ω resistor. The QHR device is mounted on a special holder inserted in a ^4^He cryostat containing a superconducting magnet and a ^3^He refrigeration system. Magnetic fields up to 16 T and temperatures as low as 0.3 K are achievable with this system. The QHR plateaus that are measured have resistances of 6453.2 Ω or 12 906.4 Ω with currents of 20 μA to 60 μA flowing through the device. The QHR device is measured against a bank of five 100 Ω resistors, immersed in an oil bath maintained at (25.000 ± 0.003) °C using a CCC bridge as shown in Fig. 18.

The NIST CCC devices developed by R. F. Dziuba and R. E. Elmquist are of the overlapped-tube type [108] with a commercial SQUID sensor to detect the ampereturn balance condition of the comparator. In order to eliminate leakage currents, the current sources are floating and optically isolated from one another [109]. A commercial nanovolt detector, D (see Fig. 18), senses the voltage difference across the resistors, and provides a feedback current through *R*_f_ and *N*_f_. The feedback current is monitored by measuring the voltage drop across *R*_f_ with an optically isolated digital voltmeter and is a measure of the difference of the resistor corrections. The QHR measurements are carried out only a few times a year, while the scaling measurements are done more frequently to monitor the banks of resistors used for customer calibrations. In 1999, a comparison at NIST between a transportable QHR system of the BIPM and the NIST QHR system agreed to within a combined relative standard uncertainty of 2 × 10^−9^, for similar measurements of a 100 Ω standard [110].

#### 2.4.2 Standard Resistors

Since January 1, 1990, the maintenance of the U.S. legal ohm has been based officially on the QHE. However, the complexity of the experiment and “odd-value” resistance of the QHR does not make it practical for the routine support of resistance measurements where comparisons are normally made on standard resistors of nominal decade values. Therefore, banks of 1 Ω, 100 Ω, and 10 kΩ standard resistors maintain the ohm between QHR measurements.

##### 2.4.2.1 1 Ω Resistors

**Fig. 19:**
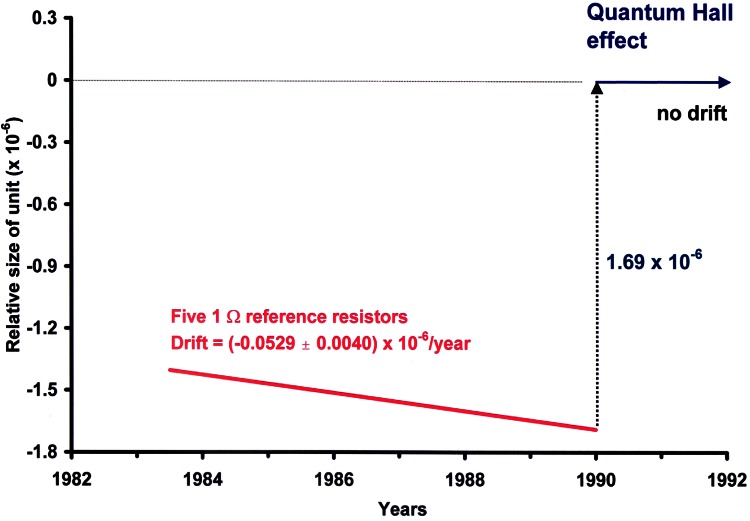
Time dependence of the U.S. ohm representation.

Thomas-type 1 Ω resistors have been used for over 50 years to maintain the laboratory value of the ohm in many NMIs, including NIST. The NIST 1 Ω bank consists of five Thomas resistors constructed in 1933 [10]; these are among a number of the original standards built by J. Thomas that are still in use. The high stability of the Thomas resistor is due to the thorough anneal at 550 °C, and the temperature coefficient of resistance (TCR) has been reduced to close to zero at a temperature between 20 °C and 30 °C by proper heat treatment. However, the Thomas resistors need to be operated in a controlled environment (oil bath) because the curvature of its temperature vs. resistance curve over this range is approximately −0.5 × 10^−6^/K^2^. The resistors are sealed in dry air in the annular space between two coaxial cylinders because the wire is subject to surface oxidation and has a significant pressure coefficient of resistance (PCR). Figure 19 indicates the drift of the 1 Ω bank prior to the re-definition of the ohm in 1990. Until recently, the Thomas-type resistors were available commercially.

##### 2.4.2.2 100 and 10 k Standard Resistors

Both the 100 Ω and 10 kΩ banks of resistors consist of commercial standard resistors hermetically sealed in oil-filled containers. Each standard contains ten 1 kΩ resistors constructed of Evanohm^1^ wire wound on mica cards and connected in parallel for the 100 Ω standards and in series for the 10 kΩ standards. The 100 Ω reference bank consists of five standards housed in an oil bath controlled at a temperature of (25.000 ± 0.003) °C. The 10 kΩ reference bank contains two standards maintained in a laboratory environment at a temperature of (23.0 ± 0.5) °C.

##### 2.4.2.3 High-Value Standard Resistors

**Fig. 20:**
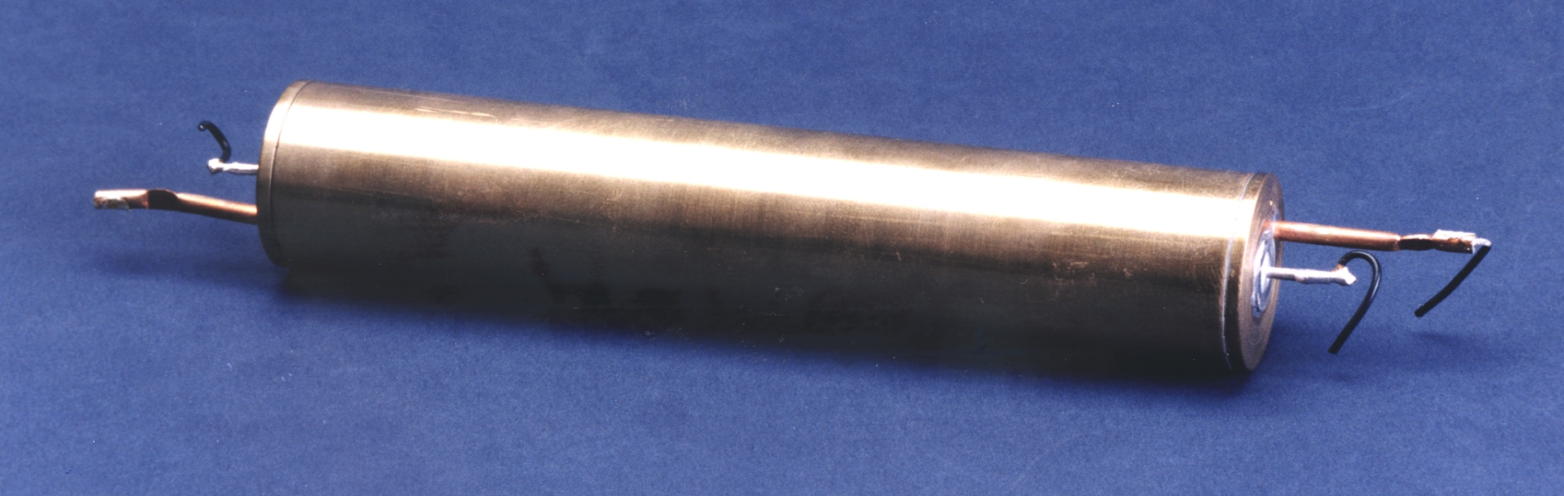
Hermetically-sealed resistor container assembly with glass-to-metal seals and copper purging tubes soldered to end plates.

In 1996, R. F. Dziuba and D. J. Jarrett developed a process for fabricating stable, transportable, high-value standard resistors of decade nominal values from 1 GΩ to 10 TΩ [111]. The resistance elements of these standards consist of precious-metal-oxide (PMO) film resistors that are commercially available. To improve their stability, these PMO film resistors are pre-aged by external heating. For the 1 GΩ, 10 GΩ and 100 GΩ standards, selected resistors are mounted in thick-walled brass cylinders using end plates with glass-to-metal seals (as shown in Fig. 20). These containers are purged with dry nitrogen gas, hermetically sealed, and shock mounted in aluminum enclosures. The shock-mounting technique reduces effects due to transport. Specially designed metal-insulator-metal containers are used to seal the 1 TΩ and 10 TΩ standards. This design allows the metallic end fittings to be driven at separate guard potentials nominally equal to the potentials at the resistor terminations and greatly suppresses leakage currents flowing across the glass insulator of the seals. Significant improvement in stability and the elimination of humidity and pressure effects is achieved by pre-aging and hermetically sealing the PMO film resistors.

#### 2.4.3 Techniques Used for Resistance Calibrations

The best evidence indicates that a Wheatstone bridge manufactured by the Otto Wolff firm in Berlin was used to compare resistance standards during the early years of NIST. It included a method for extending the resistance range by 10:1 ratios. F. Wenner designed a bridge for comparing resistors that was built in 1918 and continued in service for over 50 years [112]. It was a combination bridge that could be used as a simple Wheatstone bridge or Kelvin double bridge with a 1:1 or 10:1 ratio. In 1969, a dc current comparator bridge [113] replaced the Wenner bridge for the comparison of Thomas-type 1 Ω resistance standards. This dc current comparator measurement system was automated in 1982 and is still in use today for calibrating customer resistors. Scaling to higher resistance decades was achieved through the use of Hamon transfer standards having 10:1 and 100:1 ratios [114].

NIST provides a calibration service for standard resistors of nominal decade values from 10 ^−4^ Ω to 10^14^ Ω. To achieve low uncertainties, eight measurement systems have been developed that are optimized for the various resistance levels [115]. Over the years from 1982 to 1997, six of the systems, covering the full 19 decades of resistance, have been automated. The main methods of comparing standard resistors for NIST calibrations utilize direct current comparator (DCC) bridges and resistance-ratio bridges.

##### 2.4.3.1 Direct Current Comparator Bridges

The DCC, because of its insensitivity to lead resistances, high level of resolution, and excellent ratio linearity and ratio stability, is used to measure four-terminal standard resistors at the low end of the resistance range from 100μΩ to 100 Ω. For example, Thomas-type resistors (five comprising the reference bank, along with two check standards and eight resistors under test) are connected in series in the primary circuit of one automated DCC bridge [116]. The value of an unknown or check standard can be determined by indirectly comparing its voltage drop to the mean of the voltage drops of the reference bank via a stable 0.5 Ω resistor in the secondary circuit of the DCC. The relative standard uncertainty of this measurement system is estimated to be 0.022 × 10^−6^.

##### 2.4.3.2 10 k Measurement System Resistance-Ratio Bridge

**Fig. 21:**
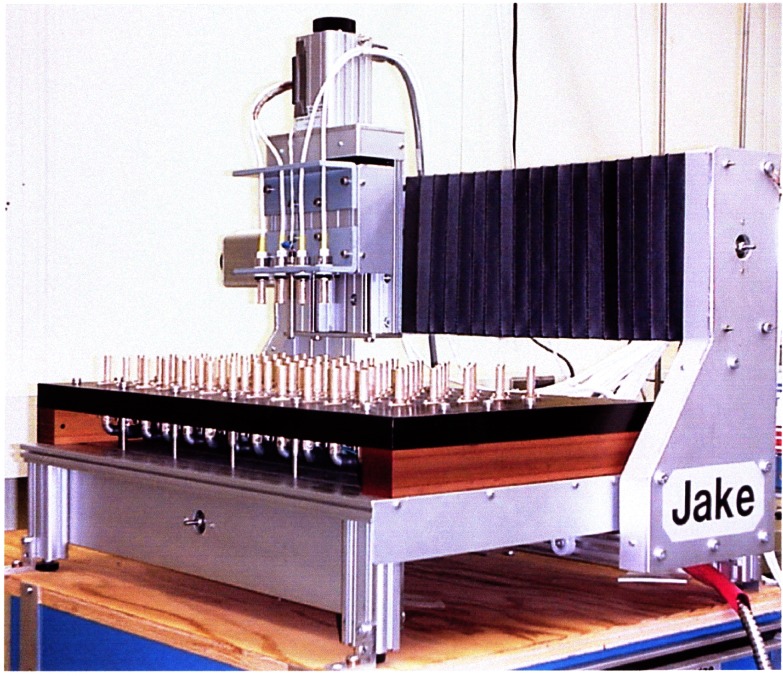
Photograph of the programmable guarded coaxial switching system. The robotic *xyz* translation stage used for switching has been labeled “Jake”.

Many industrial standards laboratories maintain their primary reference standard of resistance at 10 kΩ, near the middle of the resistance range; consequently, this resistance level constitutes a significant portion of the calibration workload at NIST. To improve these measurements an automated guarded system for the comparison of 10 kΩ standard resistors was developed [117]. The measurement system is based on the War-shawsky bridge, which includes auxiliary or fan resistors at the branch points of the bridge to eliminate first-order errors caused by lead resistances. The automatic selection of resistors is achieved by a unique, programmable, guarded coaxial-connector panel. A computer-controlled *XYZ* positioning system (shown in Fig. 21) is used to move a four-connector *Z*-axis panel (connected to the bridge) over a panel of 72 coaxial connectors mounted in the *XY* plane. This provides for 18 independent four-terminal channels. The combined relative standard uncertainty of this measurement system is 0.02 × 10^−6^.

##### 2.4.3.4 1 kΩ to 1 M Measurement System Resistance-Ratio Bridge

**Fig. 22:**
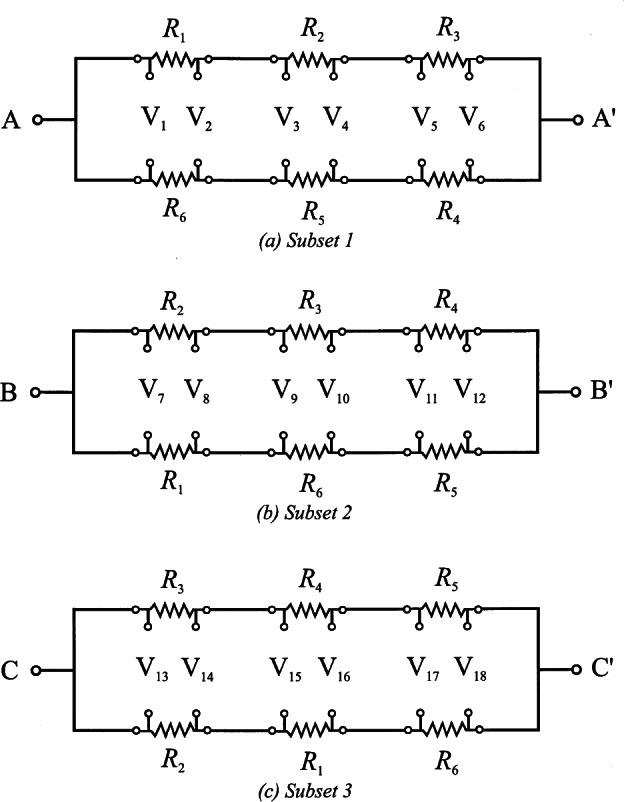
Connection of resistors for measurements by the ring method, showing the three subsets of connections for voltage measurements.

An automated measurement system, based on the unbalanced-bridge technique, was developed in 1989 to replace the manual system used to measure resistors in the range 1 kΩ to 1 MΩ [115]. This technique is re ferred to as the “ring method” since the resistors are connected in a ring configuration as shown in Fig. 22. The system is designed to measure the differences among six nominally-equal, four-terminal standard resistors of the Rosa type that are mounted on a stand located in a temperature-controlled oil bath, but it has the flexibility to accommodate resistors operating in the laboratory air environment. In operation a voltage is applied across opposite corners of the ring (A and A*'*), which divides the ring into two paralleling branches each containing three resistors. Then, a DVM measures voltages between opposite potential terminals of the resistors (V_1_ through V_6_) for the two directions of current. To complete the sequence of measurements, the applied voltage points are rotated in a clockwise or counterclockwise direction to each other pair of resistor connection points (B and B*'*) and (C and C*'*). Again voltage measurements are taken between corresponding terminals of the resistors that are at nearly equal potentials. From the three subsets of voltage measurements, one obtains a set of nine linear equations that can be solved using a least-squares technique. Values of the resistors can be calculated if the value of at least one of the resistors in the ring is known. The system is operated with two reference standards, one check standard, and three unknowns.

##### 2.4.3.5 10 MΩ to 100 TΩ Measurement System

**Fig. 23:**
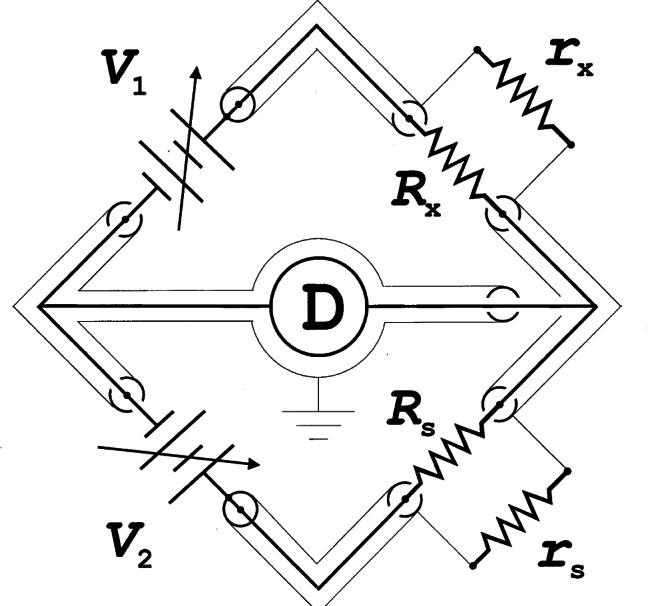
Schematic of a guarded multimegohm resistance bridge, used for comparing standard resistors up to 100 TΩ.

In 1996, an automated guarded bridge was developed for calibrating multimegohm standard resistors from 10 MΩ to 100 TΩ [118]. This innovative bridge differs from the conventional Wheatstone bridge in that two of the ratio arms are replaced by programmable voltage sources. The low output impedances of the voltage sources along with the active guard network reduce errors caused by leakage currents. As shown in Fig. 23, *R*_x_ and *R*_s_ are the unknown and standard resistors with their respective guard resistors *r*_x_ and *r*_s_. Multiple ratios up to 1000/1 can be selected by adjusting the outputs of the voltage sources. An electrometer with a resolution of ±3 fA in the current mode is used as the detector. The ratio of the guard resistors *r*_x_/*r*_s_ is nominally equal to *R*_x_/*R*_s_ and at balance, *R*_x_ = *R*_s_*V*_1_/*V*_2_.

#### 2.4.4 Resistance Scaling

NIST maintains a bank of reference standard resistors at each decade level of resistance. An unknown standard resistor is indirectly compared to a reference bank of the same nominal value using the substitution technique, where the unknown and reference resistors are sequentially substituted in the same position of a bridge circuit. This technique tends to cancel errors caused by ratio non-linearity, leakage currents, and lead and contact resistances. To verify that the values of the reference banks are consistent with the QHR, scaling measurements are completed periodically proceeding from the 1 Ω, 100 Ω or 10 kΩ banks, whose values are based on recent QHR determinations, to the other reference banks. The up or down scaling is done in steps of 10 or 100, using either a CCC bridge, Hamon transfer standards, or DCC bridge.

##### 2.4.4.1 CCC Bridge Scaling

Periodically, a CCC bridge in the calibration laboratory is used to intercompare the 1 Ω, 100 Ω, and 10 kΩ reference banks to verify the consistency of their predicted values based on previous QHR determinations. Also, these banks are checked against a 1 Ω standard resistor and 100 Ω reference bank in the QHR laboratory. All measurements are done with the resistors in situ by means of shielded cables between oil baths in the two laboratories. The automated CCC bridge system is similar to those described in Sec. 2.4.1 with a combined relative standard uncertainty of 0.005 × 10^−6^.

##### 2.4.4.2 Hamon Scaling

Hamon transfer standards provide accurate ratios of 10/1 and 100/1 for extending the resistance range in multiple decade values from 10 kΩ to 100 TΩ. The main advantage of these transfer standards is that they are calibrated at one resistance level and are then used with equal accuracy at different resistance levels as short-term reference standards. Typically, the Hamon device contains ten nominally-equal resistors permanently connected in series by means of “tetrahedral” junctions. Each junction has two current and two potential terminations, and the four-terminal resistance of each junction is adjusted to be negligible compared to the resistance of a main resistor. The transfer standard can be connected with the ten resistors in a parallel mode using special fixtures. The Hamon transfer standard can also be connected in a series-parallel configuration to establish an accurate 10/1 ratio. Hamon-type resistance scaling from 1 Ω to 100 Ω and from 100 Ω to 10 kΩ were checked against measurements with a CCC bridge and agreement was within a fractional amount of about 0.01 × 10^−6^, the practical limit of accuracy using Hamon transfer standards with conventional resistance bridges.

##### 2.4.4.3 DCC Scaling

It is difficult to construct accurate Hamon transfer standards with main resistances of less than 10 Ω. The major difficulties are with the adjustment and stability of the resistances associated with the tetrahedral junctions and fan resistors. Therefore, to extend the resistance scale below 1 Ω, NIST uses DCC resistance bridges with ratios of 1/10, 1/100, and 1/1000. The ratio accuracy and linearity of a DCC is self-checking with a resolution of better than 0.01 × 10^−6^. The ratio accuracy can also be checked using two higher-valued standard resistors whose values are based on CCC or Hamon scaling techniques.

## 3. Electrical Metrology at NIST in the Twenty-first Century

The four metrological areas we have described in the last section provide NIST customers with a sound basis for measurements of voltage, impedance, and through Ohm’s law, of current. The quantum-effect standards on which the volt and ohm are now based, the thermal voltage converter, and the calculable capacitor are some of the most fundamental advances of modern metrology. There have also been innumerable incremental advances in electrical metrology over the past hundred years, which have brought about changes that are no less significant. These include transportable reference standards used in international comparisons, which can maintain voltage and resistance values to within a fractional amount of a few times 10^−8^, digital meters with remarkably stable and linear measurement capabilities, and better techniques for measuring the fundamental electrical and mechanical quantities of power and energy. The 21st century may also provide startling new physics and metrology, and metrologists at NIST are continuing to redesign and improve the structure of basic electrical measurement standards. Meanwhile, technology is changing rapidly and improvements in telecommunications, information processing, and instrumentation are being explored as vehicles for delivering measurement services more effectively.

### 3.1 Telemetrology

The Internet and new communication technologies will influence metrology in this century, much as the telephone, fax, and email did in the last. The early stages of this influence can be seen in telemetrology projects that began at NIST in 1998.

#### 3.1.1 SIMnet

The Interamerican Metrology System (SIM) was established in 1979 to assist the 13 Latin American member countries set up and maintain NMIs. In the 1990s, SIM was expanded to include most of the countries in the Americas (now 32 members). A division of the Organization of American States, SIM consists of five geographical Metrology Regions: NORAMET (North America), CAMET (Central America), CARIMET (Caribbean), ANDIMET (Northern South America) and SURAMET (Southern South America). One of the main objectives of SIM is to harmonize the basic measurement standards in each country in the hemisphere. SIM provides a framework for international comparisons that support this objective.

SIM has sponsored international comparisons in mass, pressure, volume, and electricity in the latter half of the 1990s. These comparisons use traveling standards that are calibrated at each of the participating laboratories. The first electrical comparison was started with five digital multimeters (DMMs), calibrated at NIST (the pilot lab) in 1997, and sent to “pivot” labs in each metrology region. Each of the pivot labs then circulated the traveling standards to the NMIs within their region. Communications between metrologists in SIM were done by fax and email, which was just becoming available at most of the NMIs.

In the fall of 1998, a project began at NIST to create a communications network between the NMIs in SIM. Dubbed SIMnet [119, 120]; it is a network of computers devoted to video and data conferencing through the Internet to facilitate international comparisons, foster collaboration between metrologists, promote exchange of information, standardize test procedures, and share software and data.

Early experience with the Internet-based video conferencing software demonstrated how sensitive performance was to different hardware, software drivers, and operating systems. As a result, dedicated SIMnet video conferencing stations were designed. The stations include a desktop computer, digital camera, headset, and software to compress and transmit audio and video. The camera provides real-time video and high-quality still images, allowing small hardware details and instrument connections to be examined remotely via the Internet.

In addition to providing video conferencing capability, the station has an important advantage in international comparisons where computer-controlled instruments are often used as traveling standards. Control software is used to program instrument parameters such as range, settling time, and averaging. However, it can be difficult to verify that different control software is implementing the agreed upon test procedure. With the standard SIMnet station at each NMI, it is possible to run the same test software at each lab.

**Fig. 24:**
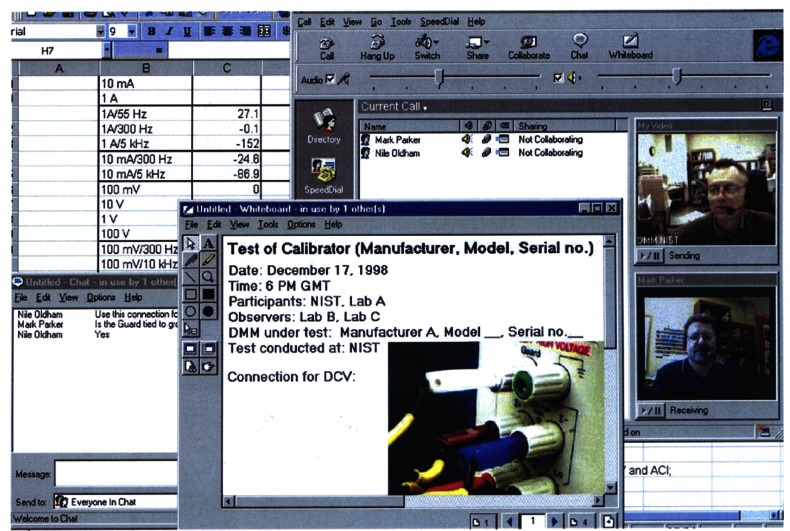
Typical video conference screen with video images of the participants on the right in the main panel, a shared spreadsheet in the upper left, the chat window in the lower left, and the whiteboard (electronic notebook) highlighted in the center.

Video conferencing tools available on the SIMnet station include the following (see Fig. 24):

*Chat* is a text communication tool that can be viewed and used by all participants in the meeting. When Internet traffic reduces audio quality, a back-up form of communication is needed. Chat is also useful in a multipoint conference for questions or comments.
*Whiteboard* is an important communication and documentation accessory. Participants can paste graphs, data, photographs, and pictures from other applications. This preserves session information in an electronic notebook available to all participants in the meeting. Unlike the real-time video, images pasted on the whiteboard are the same quality for all participants.
*Share* is a useful tool in data conferencing. One participant can share with the others the window of a currently running program, like a spreadsheet. All participants see the same window, as shown in Fig. 24.
*Collaborate* is an extension of sharing; the participants can not only observe the screen of the running application but also control it.

In December 1998 SIMnet was unveiled at NIST. Eleven other NMIs within SIM were presented with SIMnet stations and instructed in their use. To join a conference, participants log-on to the SIMnet server, which is maintained at NIST. The main task of the server is to provide audio and video to all participants in a multipoint conference. This feature is not presently available without a special server. The server sits outside the NIST network firewall and thus can be accessed by any NMI. Since its inauguration, SIMnet has been continuously tested, and in March 1999 it was used for multipoint video conferencing during the final phase of the SIM International Comparison of Electrical Units.

#### 3.1.2 Internet-Assisted Measurement Assurance Program

For many years, NIST has provided what is called a Measurement Assurance Program service (MAP) for a number of electrical quantities [81]. The MAP is designed on the principle that it is more valuable to test the customer’s calibration process, rather than the traveling standard. In a typical MAP, a NIST-owned standard is calibrated and shipped to the customer, where it is calibrated as an unknown. The standard and customer test data are then returned to NIST where a follow-up calibration and data analysis are performed. A calibration report is issued for the customer’s test system rather than just the traveling standard. Communication between NIST and the customer during the test is by telephone or email. Since the data are returned with the standard, if something is done incorrectly, the usefulness of the calibration is diminished and the standard may have to be returned to the customer for repeat measurements.

A program is evolving at NIST to allow a customer’s process to be monitored by NIST staff during the test [120, 121, 122]. It employs the Internet to improve communications, so the customer can transfer test data, download test procedures, and use NIST control software for system evaluation. As in the SIMnet process, an interactive Internet allows the customer’s “before” and “after” data to be sent electronically to NIST where the data analysis is performed. Based on experience with this project and SIMnet, almost all measurement services at NIST could utilize the capabilities of the Internet at some time in the future. Of course, most traveling electrical standards will not be replaced by code traveling on a digital network (i.e., no unit other than the second can be propagated digitally), but it appears that the Internet will greatly enhance measurement quality and efficiency.

### 3.2 The Absolute Ampere and the Quantum Age

Absolute experimental determinations of units are now known as SI realizations, and the uncertainty of the SI values of the electrical units are limited by the uncertainty of their realizations in terms of the kilogram, meter, and second. Results from the calculable capacitor experiment (Sec. 2.3) and other determinations of the fine-structure constant recently have been analytically combined [123] to yield a value of *R*_K_ with a relative standard uncertainty of 4 × 10^−9^. The Josephson constant *K*_J_ is based both on its direct measurement by voltage balances and by combining *R*_K_ with a value of the Planck constant, the latter obtained by realizing the watt in a special way. This realization of the SI watt is achieved by the moving-coil watt balance, which is a modern version of the absolute ampere experiment.

#### 3.2.1 The Moving-Coil Watt Balance

The NIST watt balance [82] has been designed to measure the ratio of mechanical to electrical power, linking the artifact kilogram, the meter, and the second to the practical realizations of the ohm and the volt derived from the QHE and the Josephson effect, respectively. Based on the equations given earlier, the Josephson voltages *U*_J_ and quantized Hall resistances *R*_H_(*i*) are linked to the atomic constants by

$$U_{J}=nf/K_{J}=\frac{nf}{2e/h},$$ (9)

$$R_{H}\left(i\right)=R_{K}/i=\frac{h/e^{2}}{i},$$ (10)

where *n* and *i* are integers, *e* is the elementary charge, *h* is the Planck constant, *f* is the frequency of the microwave radiation applied to a Josephson device, *K*_J_ is the Josephson constant, and *R*_K_ is the von Klitzing constant.

**Fig. 25:**
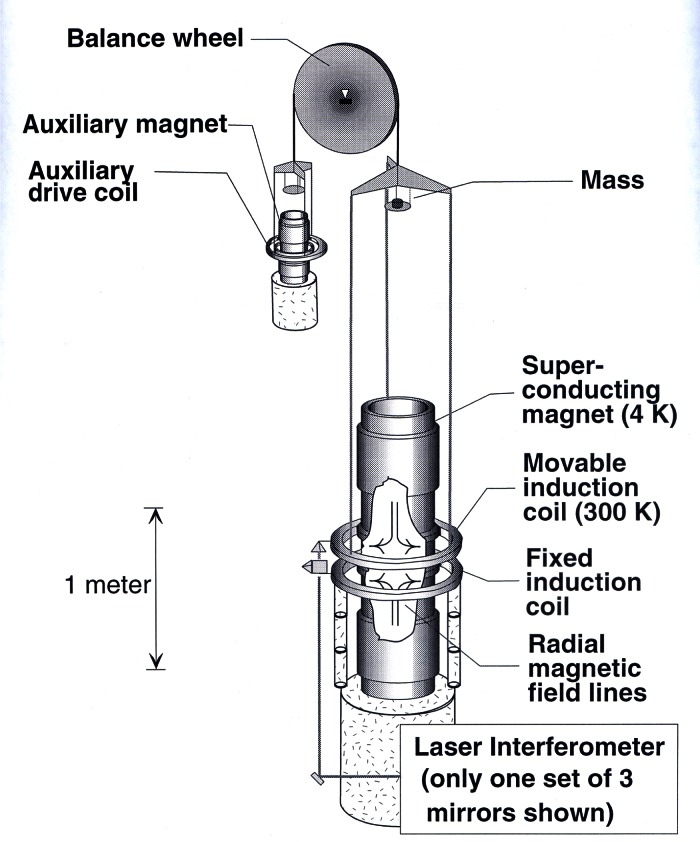
Schematic of the 1990s NIST watt balance experiment. The wheel, both magnets, and the fixed induction coil are rigidly connected. A cryostat is between the superconducting magnet and the induction coils.

The experimental method of the moving-coil watt balance, as first proposed by B. Kibble [124], consists of two measurement modes. In the first mode, a voltage reference *U* is used to servo control the velocity (d*z*/d*t*) of a coil (see Fig. 25) moving vertically in a radial magnetic flux density. In the second mode, a current *I* passing through the same coil, now held stationary in the same magnetic flux density, is used to balance the force *F_z_* = *mg*, where *m* is the mass of a standard mass and *g* is the local acceleration of gravity. The equation,

$$F_{z}/I=mg/I=-d\Phi /dz=\frac{U}{dz/dt},$$ (11)

where d*Φ*/d*z* is the vertical magnetic flux density gradient in the coil, relates the two modes. This can be rewritten as

UI=mg(dz/dt)=F(dz/dt), (12)

which equates the electric power (measured in our laboratory units) and the mechanical power (in SI units). The utilization of two separate modes of measurement is the reason that this equation can be realized with a small uncertainty. In the “velocity mode” no current flows in the moving coil and no power is dissipated. In the “balance mode”, the power dissipation from friction is negligible for the minimal motion of the balance and coil. Thus this experiment uses Eq. (12) to equate two types of virtual power, one due to gravity and one due to electrical forces.

The formal definitions for the practical units (1990 representations) of voltage *K*_J−90_ and resistance *R*_K−90_ are given in Eq. (2) and Eq. (5). Rewriting Eq. (12) to explicitly indicate the units used in the experiment, we obtain

$$\begin{array}{l} {\left\{UI\right\}}_{90}W_{90}={\left\{mg\;dz/dt\right\}}_{\text{SI}}W\;\text{or}\\ \frac{W_{90}}{W}=\frac{{\left\{mg\;dz/dt\right\}}_{\text{SI}}}{{\left\{UI\right\}}_{90}}, \end{array}$$ (13)

where *W*_90_ is the conventional unit of power based on the Josephson and quantum Hall effects and the conventional values of *K*_J−90_ and *R*_K−90_, W is the SI watt, {}_SI_ and {}_90_ mean the numerical value of the quantity in curly brackets when expressed in SI units, or 1990 practical units. Eq. (13) shows that the ratio *W*_90_/W is the direct result from observations made in the two modes of the watt experiment.

We next show that the value of certain fundamental constants are also obtained from the moving-coil watt balance experiment. Using Eqs. (9) and (10), we find *K*_J_^2^*R*_K_ = 4/*h*. From V/*K*_J_ = *V*_90_/*K*_J−90_, *R*_K_Ω = *R*_K−90_*Ω*_90_, and *W*_90_ = (*V*_90_)^2^/*Ω*90 (where *V*_90_ and *Ω*_90_ are the 1990 units of voltage and resistance) we then obtain values for *h* and *K*_J_,

$$h=\frac{4\left(W_{90}/W\right)}{K_{J-90}^{2}R_{K-90}},$$ (14)

$$K_{J}={\left(4/hR_{K}\right)}^{1/2}=K_{J-90}{\left[\left(R_{K-90}/R_{K}\right)W/W_{90}\right]}^{1/2}.$$ (15)

Watt-balance measurements of *h* and *K*_J_ do not depend on the values chosen for *K*_J−90_ and *R*_K−90_ as long as the JVS and QHE are used to measure *U* and *I* based on Eq. (13). However, *K*_J−90_ was chosen using the measurements available in 1990 to make *W*_90_/W = 1 and any measured deviation means the conventional values *K*_J−90_ and *R*_K−90_ would need to be adjusted to preserve this equality.

The first results from the NIST watt experiment, sometimes called an ampere experiment, were published in 1989 [30], giving a relative standard uncertainty for *K*_J_ of 6.7 × 10^−7^. That experiment was a prototype for the next version in which the magnetic field was increased a factor of fifty using a superconducting magnet, resulting in similar increases in the force and voltage. During the next decade many improvements were made [125, 126]. In 1998 the latest results were published [82] by E. R. Williams, R. L. Steiner, D. B. Newell, and P. T. Olsen. That work reports that *K*_J_ = 483 597.892 GHz/V with a relative standard uncertainty of 4.4 × 10^−8^ using a NIST calculable capacitor measurement of *R*_K_. This experiment provided the most accurate measurement of this quantity to date, and is described in the next section.

#### 3.2.2 A Description of the 1990s NIST Watt Experiment

Figure 25 shows the configuration of the NIST Watt experiment. The axial force on a loop of wire of radius *a* in a purely radial field, $B=[B_{a}(z)/r]\hat{r}$, where *B_a_*(*z*) is nearly constant with *z* and time, is independent of the wire shape. A superconducting magnet with 200 000 turns, consisting of two solenoid sections wound in opposition, produces a 0.1 T radial field outside the magnet cryostat. Two induction coils, each with 2355 turns, are located in the radial field. The lower induction coil is fixed to the support structure and acts as a position reference. The upper induction coil is suspended from a balance made from a pivoting wheel located above the cryostat. This allows the coil to move strictly vertically for 100 mm as the wheel rotates through an angle of ±10°. Sensors monitor the five rotational, tilting, and translational modes of motion for the induction coil, other than vertical. By using data on the coil motion along with some mutual inductance techniques, one is able to align the experiment and to estimate the alignment errors [125]. Also, the unwanted motion can be actively damped using data from these sensors.

One group of recent measurements recorded 989 values of the SI watt over a 4 month period. The total uncertainty is dominated by Type B uncertainty components, that is, components that have to be evaluated by means other than statistical analysis of repeated measurements. Of the possible Type B error sources [126] that contribute to the uncertainty, the three largest components arise from the following: (1) the index of refraction of air; (2) the present alignment procedures; and (3) residual knife-edge hysteresis effects during force measurements. Using the data discussed above Williams et al. obtained a relative standard uncertainty of 0.087 μW/W. The final result is (*W*_90_/W − 1) = (+0.8 ± 8.7) × 10^−8^, *h* = 6.626 068 91 (58) × 10^−34^ Js, and (*K*_J−90_/*K*_J_−1) = (−1.6 ± 4.4) 10^−8^.

**Fig. 26:**
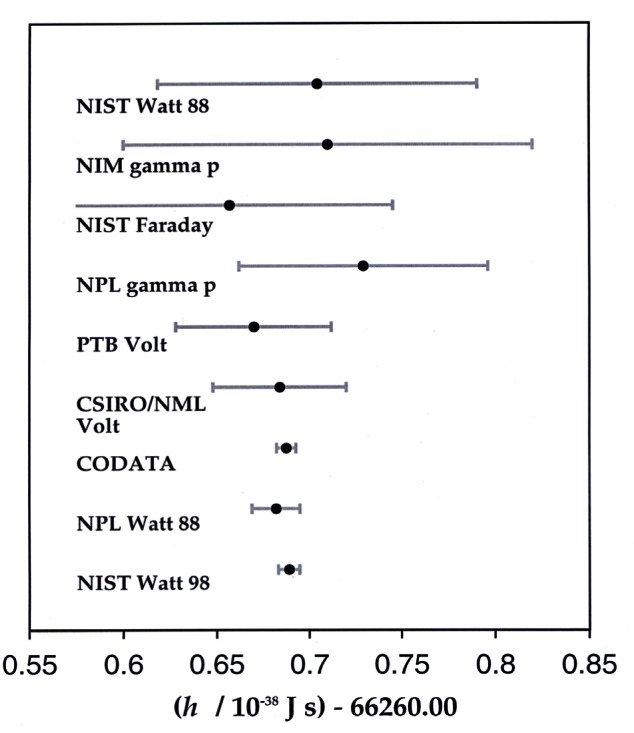
Comparison of recent electrical measurements of the Planck constant *h*. NIM: National Institute of Metrology (People’s Republic of China); NPL: National Physical Laboratory (UK); PTB: Physikalische-Technische Bundesanstalt, (Germany); CSIRO/NML: National Measurement Laboratory (Australia); CODATA: Committee on Data for Science and Technology of the International Council of Scientific Unions, Task Group on Fundamental Constants. The CODATA value of *h* is a least-squares adjusted value based on the other measurements shown here.

By connecting the macroscopic unit of mass (the kilogram) to quantum standards based on the Josephson and quantum Hall effects, this result provides a significant improvement in the Josephson constant as well as many other constants. Figure 26 compares recent measurements of the Plank constant *h*, which can be derived directly from this work with a relative standard uncertainty of 8.7 × 10^−8^.

#### 3.2.3 New Construction and Monitoring of the Kilogram

The NIST watt experiment is being completely rebuilt to achieve an improvement by a factor of ten, to less than 10 nW/W relative standard uncertainty. At that level of measurement uncertainty, the watt-balance experiment becomes a very good means of monitoring the mass artifact that is used in the weighings. The present definition of the unit of mass in the SI is based on the International Prototype of the Kilogram, which is a cylinder of platinum-iridium housed at the BIPM in France. The Prototype and a set of duplicate standards of mass accumulate contaminants on their surfaces, and must be cleaned to achieve fractional changes over the long term of less than 10^−8^ per year. Since the kilogram is the last artifact SI base unit defined in terms of a material artifact, a quantum standard of mass founded on electrical measurements would complete the modern trend of removing all artifacts from the definitions of SI units.

**Fig. 27:**
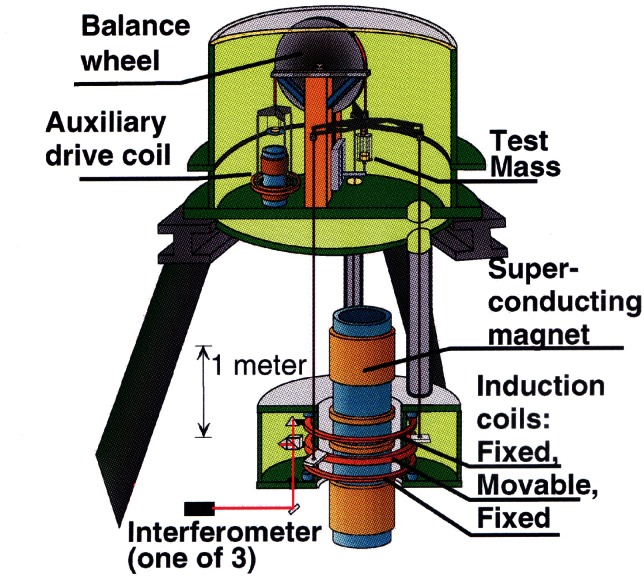
Schematic representation of the electronic kilogram apparatus. The vacuum chamber and support tripod are shown in cut-away view.

The largest uncertainties in the 1990s NIST watt experiment arose from operating in air, which required that the changing air buoyancy and refractive index be calculated from many readings of pressure, temperature, and humidity sensors. Almost every part of the balance assembly is being rebuilt to operate inside a specially constructed vacuum system consisting of two chambers, schematically represented in Figure 27. The upper chamber houses the balance section. A toroid-shaped chamber houses the inductive coils, located 3 m below and centered about the liquid helium cryostat containing the superconducting magnet.

One of the powerful aspects of the relationships in fundamental constants is that we can often derive one from a combination of others. The “electronic kilogram” experiment is designed to achieve sufficient accuracy to allow redefinition of the artifact kilogram in terms of a fundamental constant such as the Planck constant *h* or the mass of an atom such as ^12^C [127, 128]. This redefinition of mass is likely to be the last major change in the SI for many decades. Of interest here is the calculation of the mass of an atom from measurements of the moving coil watt balance. Using the theory for the Rydberg constant *R*_∞_, the following equation relates the SI mass of ^12^C to the mass used to measure the ratio *W*_90_/W in this experiment. From Eqs. (14) and (16),

$$R_{\infty }=m\left(e\right)c\alpha^{2}/2h,$$ (16)

$$m\left({\;}^{12}C\right)=\left(\frac{W_{90}}{W}\right)\left[8{\left(\frac{m\left(e\right)}{m\left({\;}^{12}C\right)}\right)}^{-1}\frac{R_{\infty }}{c\alpha^{2}K_{J-90}^{2}R_{K-90}}\right].$$ (17)

Here *c* is the speed of light, (*m*(*e*)/*m*(^12^C)) is the electron to ^12^C mass ratio, and is the fine-structure constant. The relative combined uncertainty in the group of constants inside the square bracket in Eq. (17) is below 8 × 10^−9^. At present, this watt balance experiment is the most accurate determination of *m*(^12^C) and any improvement will provide a corresponding improvement in *m*(^12^C). Thus, its measurement connects the macroscopic kilogram to the atomic mass scale.

Scientists of the Electricity Division wish to further improve the accuracy of the watt balance. If it proves possible to connect the mass of the artifact kilogram to *m*(^12^C) with accuracy equal or better than the accepted long-term stability of the artifact, then it would be time to replace the current definition for the kilogram with one based on defining an atomic mass. By defining mass in a way similar to the way time and length are now defined, relative to a Cesium hyperfine frequency (atomic clock) and the speed of light respectively, this will eliminate the last artifact standard in the SI.

## 4. Conclusion

Contributions from NIST have helped to provide absolute determinations of the values of the ampere and the ohm, and to develop quantum standards that are universal, allowing electrical quantities to be determined in units that do not change with time and that are reproducible in any laboratory. The broader achievement has been success in providing electrical standards and measurements of the finest quality. The authors would like to recognize the contributions of many not already mentioned by name, including F. B. Silsbee, F. K. Harris, B. N. Taylor, N. B. Belecki, W. D. Phillips, and all those who have built and maintained experiments, standards, and services at NIST.

## Figures and Tables

**Table 1:** Relative standard uncertainties in the measurement of the 10 pF bank with the calculable capacitor. The last row is the root-sum-square (rss) of the uncertainties listed in the rows above.

Source of uncertainty	Relative standard uncertainty (i.e., estimated relative standard deviation)
Type A standard uncertainties	

Variability of repeated observations	2 × 10^−9^

Type B standard uncertainties	

Geometrical imperfections in the calculable capacitor	15 × 10^−9^
Laser and interferometer alignment	3 × 10^−9^
Frequency (loading) corrections	4 × 10^−9^
Microphonic coupling	5 × 10^−9^
Voltage dependence	5 × 10^−9^
Transformer ratio measurement	2 × 10^−9^
Bridge linearity and phase adjustment	3 × 10^−9^
Detector uncertainties	2 × 10^−9^
Drift between calibrations and failure to close	6 × 10^−9^
Coaxial choke effectiveness	1 × 10^−9^
Temperature corrections for 10 pF capacitors	2 × 10^−9^
Relative standard uncertainty (rss)	19 × 10^−9^

## Footnotes

1 Certain commercial equipment, instruments, or materials are identified in this paper to foster understanding. Such identification does not imply recommendation or endorsement by the National Institute of Standards and Technology, nor does it imply that the materials or equipment identified are necessarily the best available for the purpose.
